# Efficacy and safety of different treatments in chemotherapy-induced thrombocytopenia: a systematic review and network meta-analysis

**DOI:** 10.3389/fphar.2025.1549214

**Published:** 2025-07-22

**Authors:** Huiyan Yang, Xiaoxiao Xu, Mei Tan, Jingyu Gao, Ruihan Fang, Xuan Liu, Zhaokun Chen, Libai Chen, Yongsheng Ruan, Yiqi Xu, Yaxin Luo, Xuedong Wu

**Affiliations:** Department of Pediatrics, Nanfang Hospital, Southern Medical University, Guangzhou, China

**Keywords:** chemotherapy-induced thrombocytopenia, efficacy, safety, network meta-analysis, randomized controlled trials, thrombopoietic agents

## Abstract

**Background:**

Chemotherapy-induced thrombocytopenia (CIT) is a challenge in cancer treatment, increasing bleeding risks and reducing chemotherapy dose. We sought to compare the efficacy and safety of various treatments for CIT.

**Methods:**

Randomized controlled trials (RCTs) involving CIT treatments were subjected to a systematic review and network meta-analysis. PubMed, Embase, Web of Science, Cochrane Library, and ClinicalTrials.gov databases were searched up to 2 July 2024.

**Results:**

Sixteen RCTs (*n* = 1,746) were included in this study. Pairwise meta-analysis showed thrombopoietic agents significantly reduced platelet transfusions (OR = 0.50; 95% CI: 0.32–0.77), improved nadir platelet count (SMD = 0.39; 95% CI: 0.25–0.53) and promoted platelet recovery ≥100 × 10^9^/L (SMD = −0.48; 95% CI: −0.68 to −0.28). Thrombopoietin receptor agonists (TPO-RAs) reduced chemotherapy delays or dose reductions (OR = 0.37; 95% CI: 0.20–0.67) and the incidence of grade 3/4 thrombocytopenia (OR = 0.50; 95% CI: 0.27–0.93). Network meta-analysis indicated that eltrombopag ranked first in reducing chemotherapy dose reductions or delays and increasing nadir platelet count. In terms of reducing the incidence of grade 3/4 thrombocytopenia, recombinant human thrombopoietin (rhTPO) ranked highest, followed by eltrombopag. Recombinant human interleukin-11 (rhIL-11) had the lowest platelet transfusion rate but the highest incidence of adverse events, whereas avatrombopag had the lowest rate of adverse events and thromboembolism. Additionally, avatrombopag outperformed eltrombopag in promoting hemoglobin and neutrophils recovery.

**Conclusion:**

Thrombopoietic agents may benefit CIT patients. TPO-RAs, particularly eltrombopag, show superior efficacy and good tolerability. Although rhIL-11 and rhTPO can rapidly promote platelet recovery and reduce platelet transfusions, they have several limitations.

**Systematic Review Registration:**

https://inplasy.com/inplasy-2024-11.0105/

## 1 Introduction

Chemotherapy-Induced Thrombocytopenia (CIT) is a common hematologic complication resulting from chemotherapy, typically resulting from the suppression of bone marrow megakaryocyte generation and function by chemotherapeutic agents, leading to a significant reduction in platelet count (Wu et al., 2009). Platelet count below 100 × 10^9^/L is diagnostic of thrombocytopenia, which, if severe, can cause bleeding tendencies, increased infection risk, and interfere with the overall treatment plan. While platelet transfusions are often used to manage severe thrombocytopenia, they carry the risk of transfusion-related adverse reactions and may lead to transfusion refractoriness (Mkhitaryan et al., 2019).

Thrombopoietic agents effectively reduce bleeding risk and minimize platelet transfusions in the management of CIT. Research suggests that these agents help maintain Relative Dose Intensity (RDI) and may extend overall survival (Schiffer et al., 2018). The main agents include recombinant human thrombopoietin (rhTPO), thrombopoietin receptor agonists (TPO-RAs) and recombinant human interleukin-11 (rhIL-11), which promote platelet production by stimulating megakaryocyte progenitor cells or specifically binding to the thrombopoietin (TPO) receptor, regulating megakaryocyte proliferation, differentiation, and maturation. rhTPO binds to the TPO receptor’s extracellular domain, inducing a conformational change and activating downstream signaling pathways, including JAK/STAT, RAS/MAPK, and PI3K/AKT, thereby promoting the development of hematopoietic stem cells and megakaryocytes, and enhancing platelet production (Varghese et al., 2017). TPO-RAs bind to the transmembrane domain of the human TPO receptor, triggering a signaling cascade that induces the proliferation and differentiation of myeloid progenitors and megakaryocytes (Quintino et al., 2021). IL-11 is used to treat grade 3/4 thrombocytopenia following chemotherapy for solid tumors and non-myeloid leukemia. However, its clinical application is constrained by side effects such as arrhythmias, fluid retention, and pulmonary edema (Gordon et al., 1996). While rhTPO is approved for CIT treatment only in China, TPO-RAs are not approved for CIT in any country due to insufficient evidence. The NCCN guidelines recommend romiplostim for CIT management (Al-Samkari et al., 2021). In terms of adverse events, thrombosis and embolism are concerns, as studies have shown that TPO-RAs may promote platelet activation in immune thrombocytopenic purpura (ITP) patients by increasing platelet microparticle formation and upregulating platelet glycoprotein VI (GPVI) and P-selectin expression (van Dijk et al., 2021).

Given the differences in target sites, in metabolism, and efficacy among various thrombopoietic agents, as well as the challenges of conducting head-to-head randomized controlled trials (RCTs) to determine the optimal treatment, we conducted a systematic review and network meta-analysis. By comparing eligible RCT data both directly and indirectly, we evaluated the efficacy, safety, and ranking of treatments, providing valuable insights for selecting the most effective treatment for CIT.

## 2 Materials and methods

### 2.1 Search strategy and study selection

Following the PRISMA Extension guidelines, this network meta-analysis was registered with INPLASY (INPLASY2024110105). A Bayesian method was used to perform the network meta-analysis. Two researchers independently searched PubMed, Embase, Web of Science, ClinicalTrials.gov, and Cochrane Central Register of Controlled Trials databases for potentially eligible studies up to 2 July 2024. Supplementary Table S1 provides a detailed description of the search strategy. Randomized controlled trials were included if they met the following inclusion criteria: cancer patients who developed CIT were enrolled, and among these patients, treatments with rhTPO, rhIL-11, modified interleukin-11 (mIL-11), and TPO-RAs (romiplostim, eltrombopag, and avatrombopag) were administered. We also included the latest conference abstracts. Non-English articles were excluded. The titles and abstracts were initially screened, followed by a review of the full texts of potentially eligible studies meeting the inclusion criteria.

### 2.2 Data extraction and risk-of-bias assessment

Key trial information, including author, publication year, patient numbers, treatments, and outcomes, was collected. In addition to platelet transfusion, the following indicators were also considered as efficacy outcomes: grade 3/4 thrombocytopenia, platelet recovery to ≥100 × 10^9^/L, bleeding events, dose delays or reductions due to thrombocytopenia, time of platelet recovery to ≥100 × 10^9^/L, nadir platelet count and incidence of neutropenia and anemia. Safety data included thromboembolic events and adverse events assessed by Common Terminology Criteria for Adverse Events (CTCAE).

The Cochrane Risk of Bias Tool was used to evaluate randomization, allocation concealment, blinding, incomplete data, selective reporting, and other biases (Cumpston et al., 2019). Trials were classified as low, high, or unclear risk of bias. Trial inclusion, data extraction, and bias assessment were conducted independently by two researchers, with final decisions made through joint deliberation.

### 2.3 Statistical analysis

The efficacy and safety of treatments for CIT were compared by synthesizing all available direct and indirect evidence, using odds ratios (OR), mean differences (MD), and corresponding 95% credible intervals (CrI) as reported outcomes. Treatment rankings were assessed using the surface under the cumulative ranking curve (SUCRA).

Using Stata (17.0), network plots were generated to illustrate the direct and indirect comparisons of various treatments. For head-to-head comparisons involving two or more treatments, paired meta-analyses were conducted. Heterogeneity was assessed using *I* ^2^ values, classified as low (<25%), medium (25%–50%), or high (>50%) heterogeneity (Higgins et al., 2003). Analyses were conducted with R software (4.3.2).

A Bayesian network meta-analysis was conducted using a Markov chain Monte Carlo simulation with the Gemtc package in R software. We used a fixed-effects consistency model (Dafni et al., 2019) and non-informative uniform and normal prior distributions. 20,000 iterations were generated for each outcome, including 5,000 burn-ins and a thinning interval of 1. Convergence was assessed using trace plots and the Brooks-Gelman-Rubin statistic in the Supplementary Figure S1, and was reached once a stable equilibrium distribution. After confirming convergence, the model parameters’ posterior distributions were obtained.

Within a Bayesian framework, the network meta-analysis ranks the treatments by Computing SUCRA, with scores ranging from 0 to 1, where 1 indicates the best treatment.

We employed the OR and MD with 95% CI, using a fixed-effects model as a conservative estimate. To evaluate global inconsistency, we compared the fit between the consistency and inconsistency models. Publication bias was assessed using a comparison-adjusted funnel plot.

Additionally, a sensitivity analysis was conducted to evaluate the robustness and reliability of the results. All outcomes with high heterogeneity were analyzed using a random-effects model with conservative prior distributions (τ^2^ ∼ Uniform (0, 5)). We performed sensitivity analyses using random-effects models through systematically excluding specific study types to evaluate the heterogeneity across studies.

## 3 Results

### 3.1 Study selection and characteristics

**FIGURE 1:**
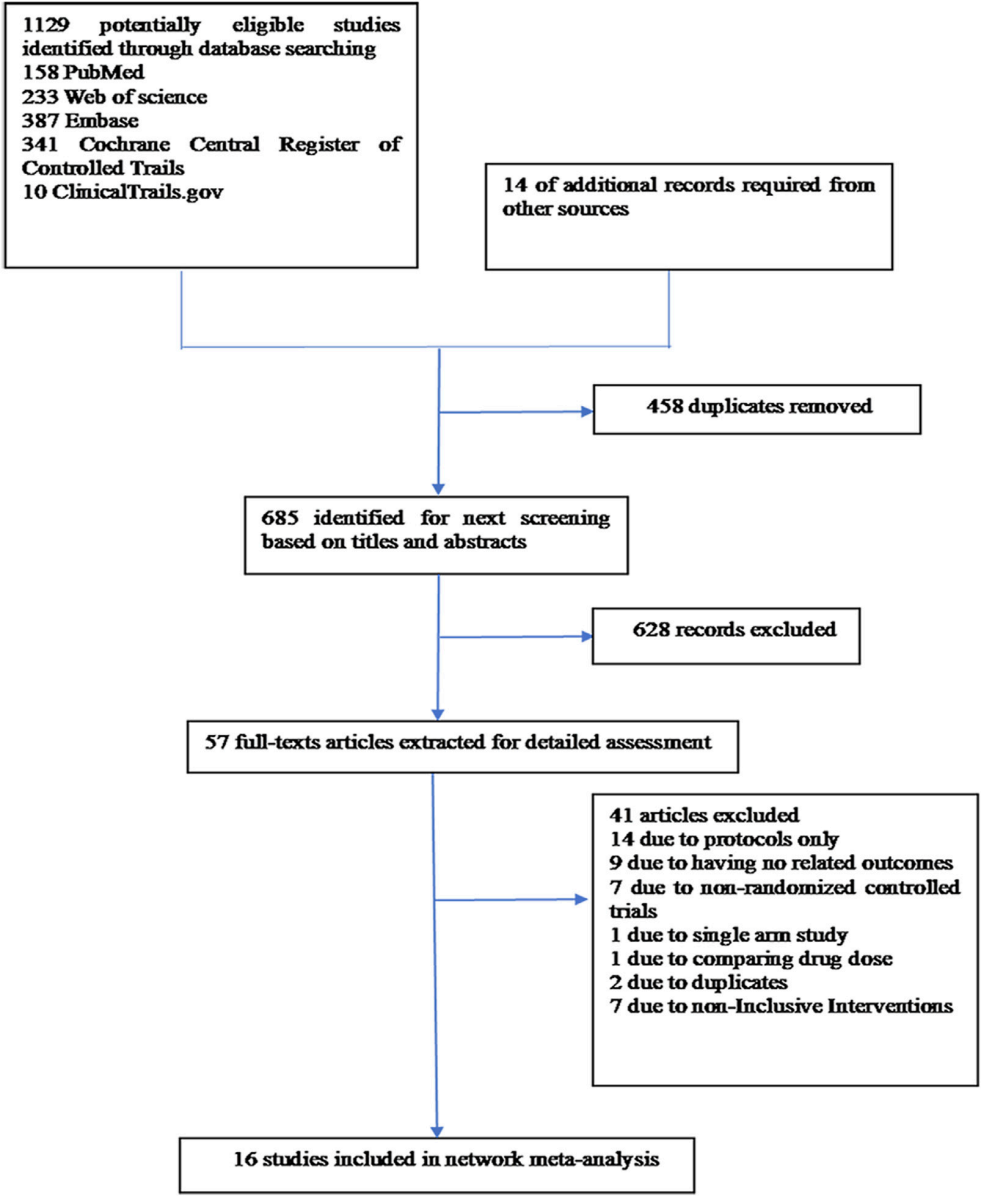
PRISMA flow diagram of the screening and selection process used in the study.

**FIGURE 2:**
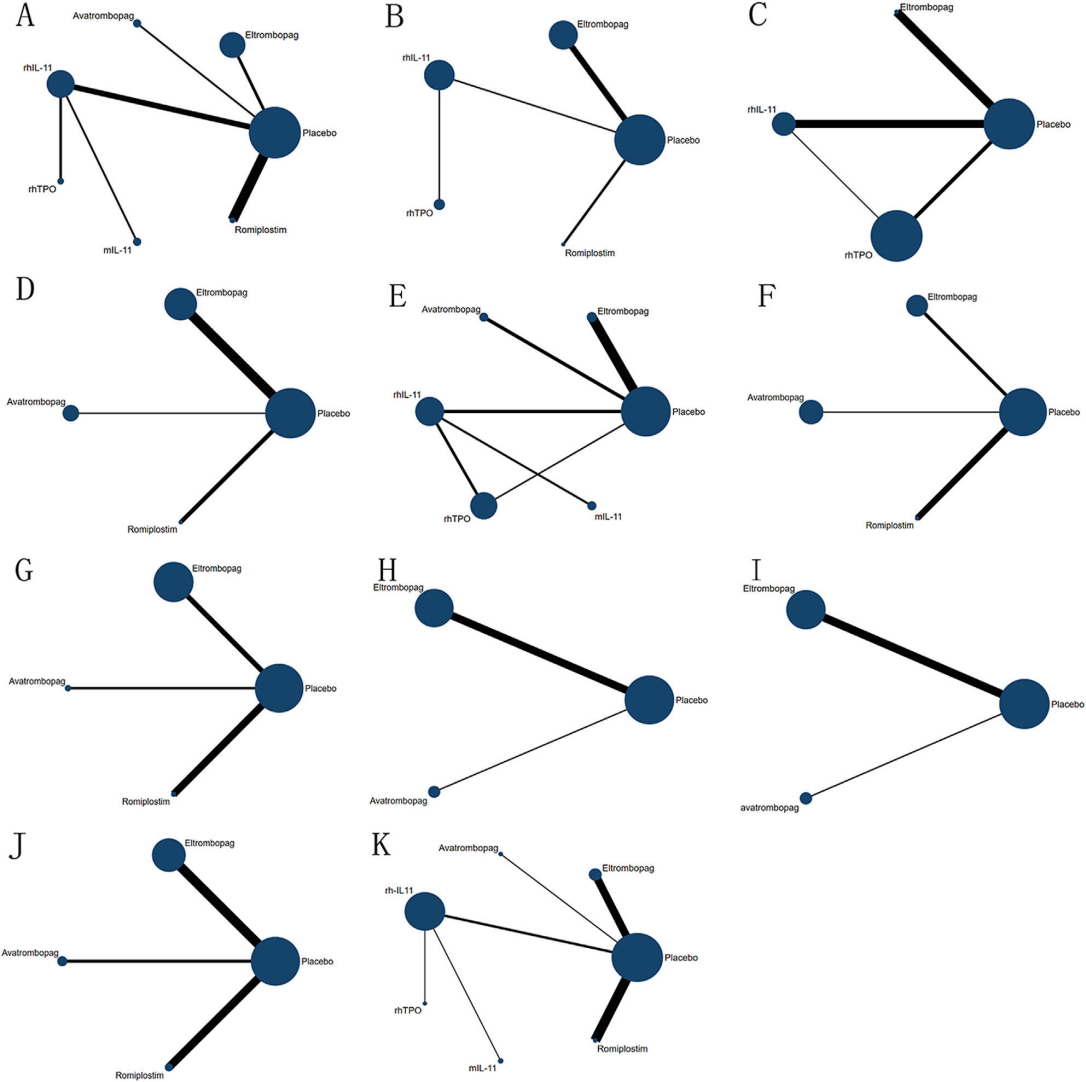
Network diagram of comparison on different outcomes in different treatment groups for patients with CIT. **(A)** Comparison of network diagrams for platelet transfusion. **(B)** Comparison of network diagrams for grade 3/4 thrombocytopenia. **(C)** Comparison of a network diagram for time of PLT recovery to ≥100 × 10^9^/L (d). **(D)** Comparison of a network diagram for PLT recovery to ≥100 × 10^9^/L. **(E)** Comparison of a network diagram for nadir platelet count. **(F)** Comparison of a network diagram for dose delays/dose reductions/missed doses. **(G)** Comparison of a network diagram for bleeding event. **(H)** Comparison of a network diagram for anemia. **(I)** Comparison of a network diagram for neutropenia. **(J)** Comparison of a network diagram for thrombosis. **(K)** Comparison of a network diagram for adverse events. rhTPO, recombinant human thrombopoietin; rhIL-11, recombinant human interleukin-11; mIL-11, modified interleukin-11.

A thorough search of each database retrieved 1,143 potential articles, from which 57 studies were selected for full-text review after the initial screening of titles and abstracts. Finally, the network meta-analysis included 16 RCT studies. The study selection flowchart is presented in Figure 1. Sixteen trails were included as follows: 4 studies with eltrombopag (Winer et al., 2017; Frey et al., 2019; Winer et al., 2015; Kellum et al., 2010), 1 with avatrombopag (Al-Samkari et al., 2022), 4 with rhIL-11 (Sun et al., 2002; Tepler et al., 1996; Isaacs et al., 1997; Zhou et al., 2016), 3 with rhTPO(Zhou et al., 2016; Bai et al., 2004; Xu et al., 2018; Chen et al., 2010), 1 with mIL-11 (Wu et al., 2012), and 3 with romiplostim (Soff et al., 2019; Greenberg et al., 2012; Tian and Jamieson, 2020). In all RCTs, a placebo was used as the control, except in six studies where rhIL-11 and a blank were used as controls. In the statistical analysis, we classified the blank group into the placebo group. The study included 1,746 patients, with 998 assigned to the experimental group and 748 to the control group. Relevant information is presented in Table 1, and the network diagram can be found in Figure 2.

**TABLE 1:** Baseline characteristics of patients and studies included that met the eligibility criteria for assessment.

Study id (phase, ethnicity)	Participants (I/C)	Female (I/C)	Age (y), median (I/C)	Tumor type	CIT intervention	Comparison	Baseline PLT count/median (×10^9^/L) (I/C)
Frey et al. (2019) (2, multiple)	74/74	38/31	56.7/56.6	AML	Eltrombopag	Placebo	59.5/63.7
Winer et al. (2017) (2, multiple)	52/23	23/13	67.0, 67.5/64.0, 66	Solid tumors	Eltrombopag	Placebo	NM/NM
Al-Samkari et al. (2022) (3, White and Asian)	82/40	43/22	61/60.8	Non-haematological malignancies	Avatrombopag	Placebo	86.1/31
Sun et al. (2002)	107/107	NG	NG	NG	rhIL-11	Placebo	136.46/246.49
Chen et al. (2010)	32/30	NG	NG	NG	rhTPO	rhIL-11	35.27/NG
Zhou et al. (2016) (China)	46/58	15/22	5/5	ALL	rhIL-11	Blank	NG
Winer et al. (2015) (1, multiple)	19/7	10/4	53, 69/55, 67.5	Solid tumors	Eltrombopag	Placebo	129/NG
Bai et al. (2004)	154/154	NG	NG	Solid tumor	rhTPO	Blank	64.4/NG
Wu et al. (2012) (2, multiple)	73/80	NG	54.7/57.7	Solid tumor	mIL-11	rhIL-11	62.6/189.6
Amgen Inc. 2009 (2, 62 white, 1 black)	51/12	NG	63.8, 62.5, 65.4/59.8	Non-small cell lung cancer	Romiplostim	Placebo	NG
Xu et al. (2018) (2, multiple)	77/31	21/13	58.7/60.8	Non-small cell lung cancer	rhTPO	rhIL-11	61.8/265.27
Greenberg et al. (2012) (2, multiple)	15/14	7/3	68/72	MDS	Romiplostim	Placebo	NG
Isaacs et al. (1997) (80% White)	40/37	NG	47.9/45.7	Breast cancer	rhIL-11	Placebo	NG
Tepler et al. (1996) (multiple)	27/27	18/15	45/46	Solid tumor	rhIL-11	Placebo	NG
Kellum et al. (2010) (2,141 white)	134/46	71/30	58.5, 59,58/58	Solid tumors	Eltrombopag	Placebo	41.6/407.3
Soff et al. (2019) (2, multiple)	15/8	10/2	50/67	Nonhematologic cancer	Romiplostim	Blank	NG/141

Abbreviations: NG, not given; AML, acute myeloid leukemia; ALL, acute lymphoblastic leukemia; rhTPO, recombinant human thrombopoietin; rhIL-11, recombinant human interleukin-11; mIL-11, modified interleukin-11. PLT, platelet count.

### 3.2 Risk of bias

The assessments of the risk of bias are summarized in the Supplementary Figure S2. Most of these RCTs exhibit a high risk in areas such as participant and outcome assessment blinding, personnel blinding, selective reporting, and incomplete outcome data. Twelve studies did not provide sufficient details on random sequence generation and allocation concealment, resulting in an unclear classification. All studies were considered to have a low risk of other bias. Five RCTs were identified as having a high risk of incomplete outcome data.

### 3.3 Outcomes

#### 3.3.1 Pairwise meta-analysis in CIT patients

**FIGURE 3:**
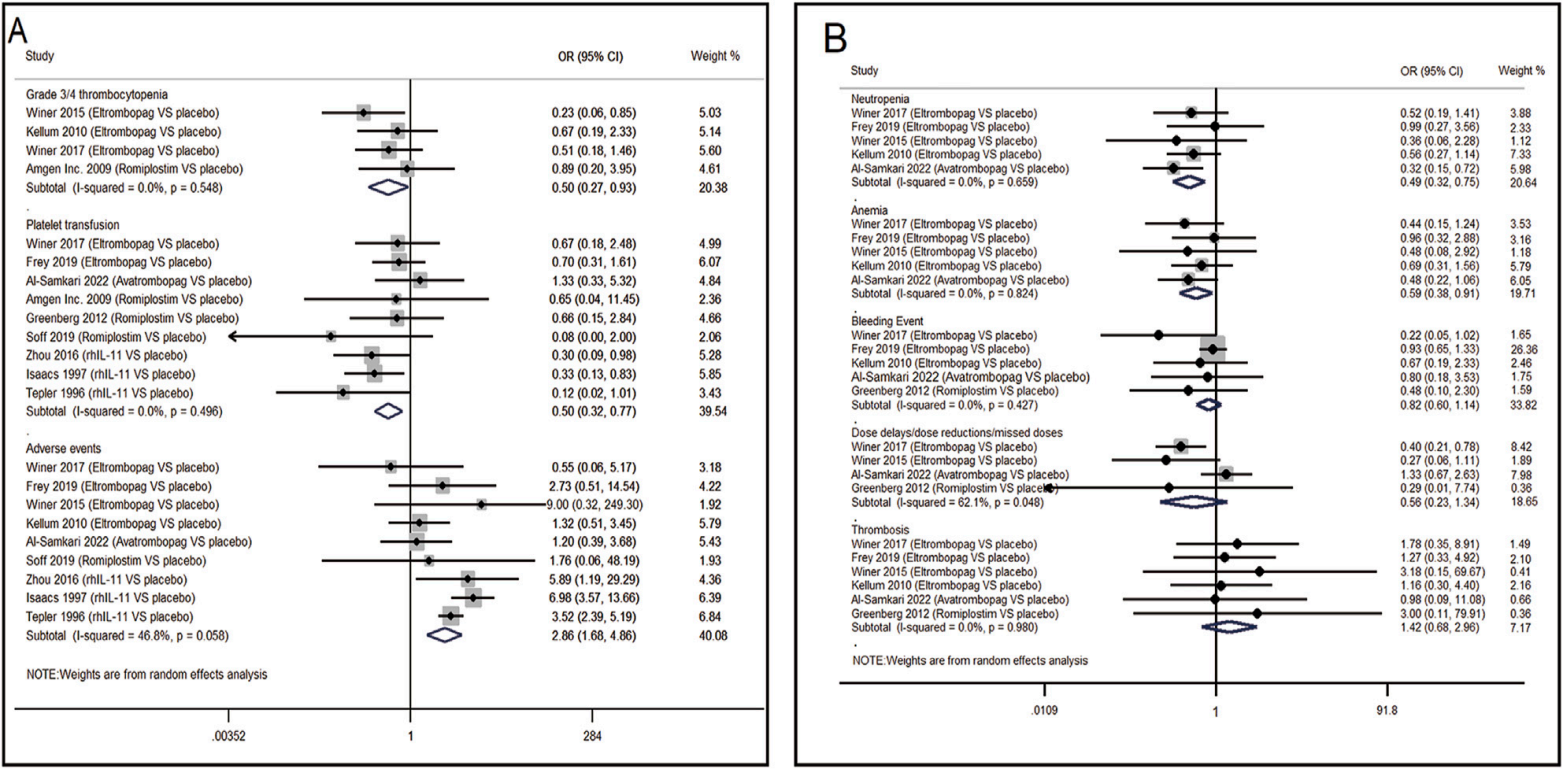
Meta-analysis of comparison of different treatments on eight indicators in CIT. **(A)** Pooled odds ratio (OR) of grade 3/4 thrombocytopenia, platelet transfusion and adverse events. **(B)** Pooled odds ratio (OR) of neutropenia, anemia, bleeding event, dose delays/dose reductions/missed doses and thrombosis in comparison of TPO-RAs versus placebo. CI, confidence interval; OR, odds ratio; TPO-RAs, thrombopoietin receptor agonists; rhIL-11, recombinant human interleukin-11.

**FIGURE 4:**
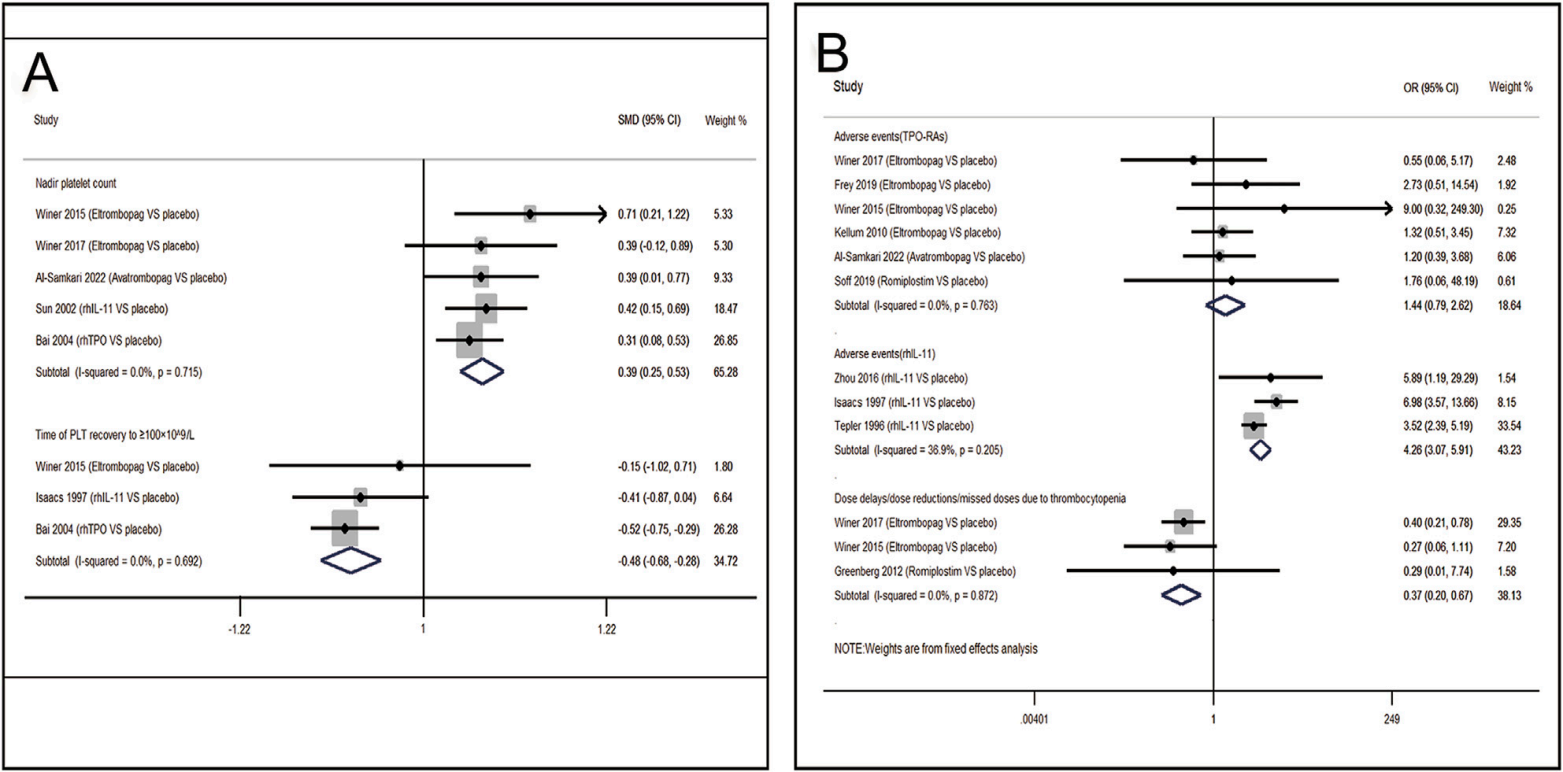
Meta-analysis of comparison of different treatments on four indicators in CIT. **(A)** Pooled odds ratio (OR) of nadir platelet count and time of PLT recovery to ≥100 × 10^9^/L in comparison of thrombopoietic agents versus placebo. **(B)** Pooled odds ratio (OR) of dose delays/dose reductions and adverse events through sensitivity analysis. CI, confidence interval; OR, odds ratio; rhTPO, recombinant human thrombopoietin; rhIL-11, recombinant human interleukin-11.

13 RCTs which were included to directly compare the therapeutic regimens (eltrombopag, avatrombopag, rhIL-11, rhTPO and romiplostim) vs. placebo, involved 1,423 patients. Thrombopoietic agents were evaluated as treatments for CIT through pairwise meta-analysis using head-to-head data. The percentage of patients needing platelet transfusions were lower in the thrombopoietic agent group compared to the placebo group (21.5% vs. 32.8%, OR = 0.50; 95% CI: 0.32–0.77) (*I* ^2^ = 0.0%, p = 0.496) (Figure 3A). The thrombopoietic agent group also demonstrated a higher nadir platelet count than the placebo group (SMD = 0.39; 95% CI: 0.25–0.53) (*I* ^2^ = 0.0%, p = 0.715) and achieved faster recovery of platelet counts ≥100 × 10^9^/L (SMD = −0.48; 95% CI: −0.68 to −0.28) (*I* ^2^ = 0.0%, p = 0.692) (Figure 4A). For grade 3/4 thrombocytopenia, the TPO-RAs group showed a lower incidence compared to placebo (21.4% vs. 34.7%, OR = 0.50; 95% CI: 0.27–0.93) (*I* ^2^ = 0.0%, p = 0.548) (Figure 3A). No significant difference in bleeding incidence was observed between groups. Additionally, TPO-RAs reduced the occurrence of neutropenia (OR = 0.49; 95% CI: 0.32–0.75) (*I* ^2^ = 0.0%, p = 0.659) and anemia (OR = 0.59; 95% CI: 0.38–0.91) (*I* ^2^ = 0.0%, p = 0.824) (Figure 3B). Further sensitivity analysis by removing the high-heterogeneity study by Al-Samkari revealed that the TPO-RAs group had significantly fewer patients with chemotherapy dose reductions or delays due to thrombocytopenia than the placebo group (OR = 0.37; 95% CI: 0.20–0.67) (*I* ^2^ = 0.0%, p = 0.872). Through subgroup analysis, there were similar rates of adverse events between the TPO-RAs and placebo groups, but rhIL-11 was higher than placebo (OR = 4.26; 95% CI: 3.07–5.91; p > 0.05) (Figure 4B).

#### 3.3.2 Network meta-analysis in CIT patients

**FIGURE 5:**
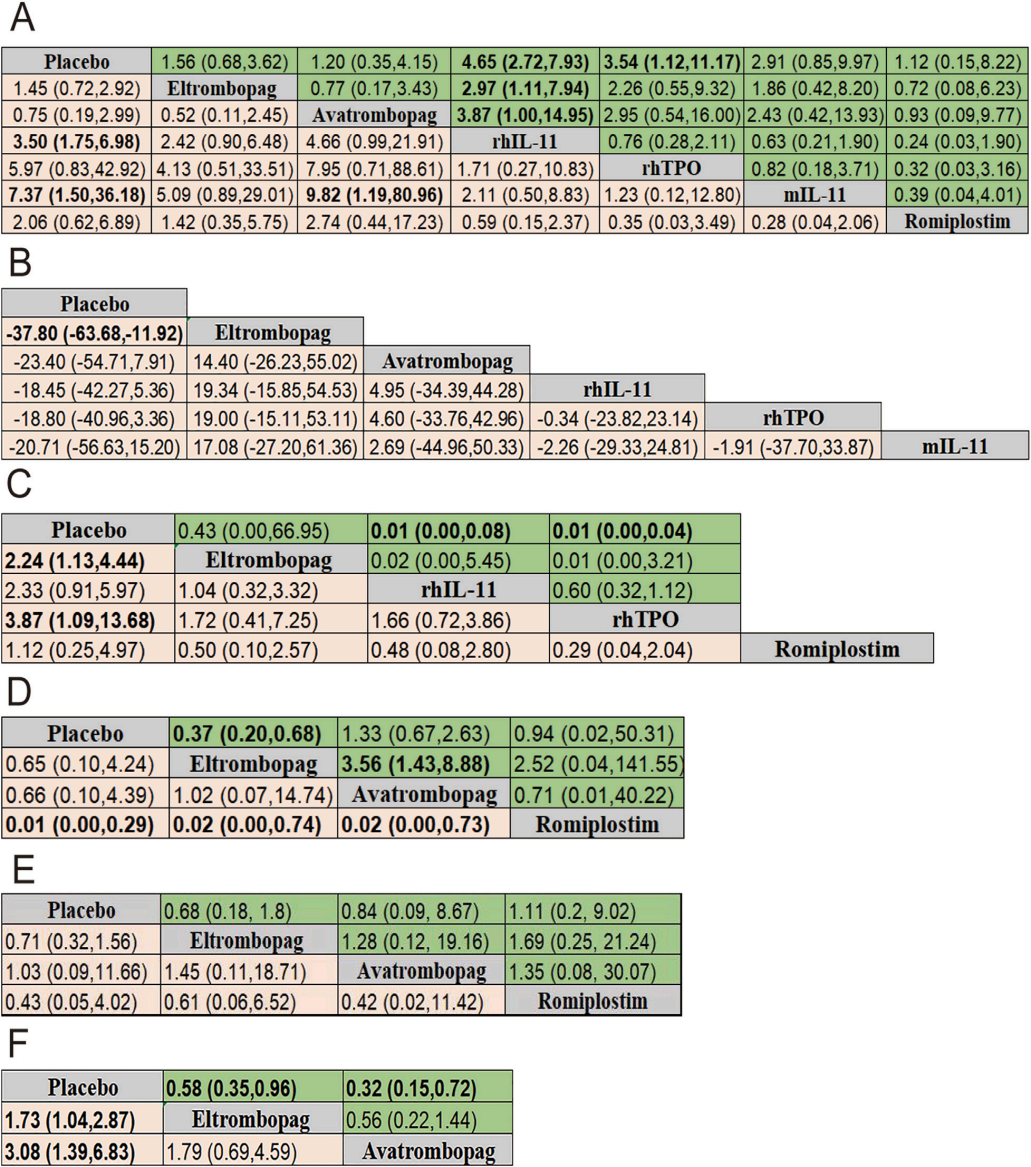
Pooled estimates of the network meta-analysis. **(A)** Pooled ORs (95% credible intervals) for adverse events in the upper triangle and platelet transfusion in the lower triangle. **(B)** SMD (95% credible intervals) for nadir platelet count. **(C)** SMD (95% credible intervals) for time of PLT recovery to ≥100 × 10^9^/L (d) in the upper triangle and Pooled ORs (95% credible intervals) for grade 3/4 thrombocytopenia in the lower triangle. **(D)** Pooled ORs (95% credible intervals) for dose delays/dose reductions in the upper triangle and PLT recovery to ≥100 × 10^9^/L in the lower triangle. **(E)** Pooled ORs (95% credible intervals) for bleeding event in the upper triangle and thrombosis in the lower triangle. **(F)** Pooled ORs (95% credible intervals) for neutropenia in the upper triangle and anemia in the lower triangle. Data in each cell is OR (95% CrIs) for the comparison of row-defining treatment versus column-defining treatment. OR greater than 1 favor upper-row treatment. Significant results are highlighted in bold. OR, odds ratio; rhTPO, recombinant human thrombopoietin; rhIL-11, recombinant human interleukin-11; mIL-11, modified interleukin-11.

The results of efficacy and safety endpoints from network meta-analysis are shown in the ladder diagram (Figure 5). rhIL-11 and mIL-11 needed lower platelet transfusions (placebo vs. rhIL-11 [OR = 3.50; 95% CI: 1.75–6.98]; placebo vs. mIL-11 [OR = 7.37; 95% CI: 1.50–36.18]). mIL-11 group also showed a significantly reduced rate of transfusions compared to the avatrombopag group (avatrombopag vs. mIL-11 [OR = 9.82; 95% CI: 1.19–80.96]). For grade 3/4 thrombocytopenia, eltrombopag and rhTPO reduced the incidence compared to placebo (placebo vs. eltrombopag [OR = 2.24; 95% CI: 1.13–4.44]; placebo vs. rhTPO [OR = 3.87; 95% CI: 1.09–13.68]). Eltrombopag demonstrated a clear advantage over placebo in reducing chemotherapy delays/reductions and increasing nadir platelet count (eltrombopag vs. placebo [OR = 0.37; 95% CI: 0.20–0.68]; placebo vs. eltrombopag [SMD = −37.80; 95% CI: −63.68 to −11.92]). In terms of the percentage of patients achieving a platelet count ≥100 × 10^9^/L, the romiplostim group showed significantly higher rates compared to the eltrombopag, avatrombopag, and placebo groups, with pooled OR values of 0.02 (95% CI: 0.00–0.74), 0.02 (95% CI: 0.00–0.73), and 0.01 (95% CI: 0.00–0.29), respectively. Additionally, both rhIL-11 and rhTPO groups demonstrated a significantly faster platelet recovery to ≥100 × 10^9^/L compared to placebo (rhIL-11 vs. placebo [SMD = 0.01; 95% CI: 0.00–0.08]; rhTPO vs. placebo [SMD = 0.01; 95% CI: 0.00–0.04]). No significant differences in bleeding incidence among placebo, eltrombopag, avatrombopag, and romiplostim.

For reducing the incidence of neutropenia and anemia, both eltrombopag and avatrombopag were significantly more effective than placebo (placebo vs. eltrombopag [OR = 1.73; 95% CI: 1.04–2.87]; placebo vs. avatrombopag [OR = 3.08; 95% CI: 1.39–6.83]) for neutropenia, and eltrombopag vs. placebo [OR = 0.58; 95% CI: 0.35–0.96]; avatrombopag vs. placebo [OR = 0.32; 95% CI: 0.15–0.72] for anemia).

A total of 13 studies were included to analyze adverse events (AEs) related to each intervention or potentially related, with all possible pairwise comparisons performed. Results showed that rhIL-11 had the highest AEs risk compared to placebo (OR = 4.65; 95% CI: 2.72–7.93), followed by rhTPO (OR = 3.54; 95% CI: 1.12–11.17). Compared with eltrombopag and avatrombopag, rhIL-11 also showed higher AEs risks ([OR = 2.97; 95% CI: 1.11–7.94] [OR = 3.87; 95% CI: 1.00–14.95] respectively). No significant differences were observed in adverse events with the remaining four drugs. Additionally, seven studies compared thrombosis incidence among interventions, finding no significant differences.

#### 3.3.3 Rank probabilities

**FIGURE 6:**
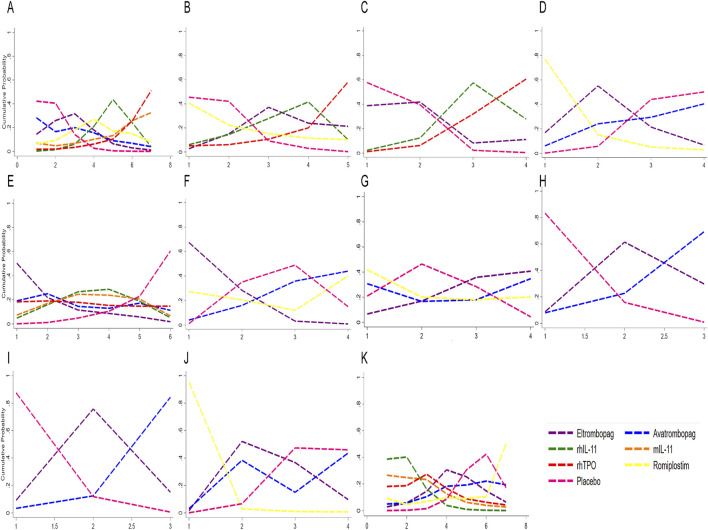
Profiles indicate the probability of each comparable treatment being ranked from first to last. **(A)** Sucra for platelet transfusion. **(B)** Sucra for grade 3/4 thrombocytopenia. **(C)** Sucra for time of PLT recovery to ≥100 × 10^9^/L (d). **(D)** Sucra for PLT recovery to ≥100 × 10^9^/L. **(E)** Sucra for nadir platelet count. **(F)** Sucra for dose delays/dose reductions. **(G)** Sucra for bleeding event. **(H)** Sucra for anemia. **(I)** Sucra for neutropenia. **(J)** Sucra for thrombosis. **(K)** Sucra for adverse events. rhTPO, recombinant human thrombopoietin; rhIL-11, recombinant human interleukin-11; mIL-11, modified interleukin-11.

The ranking of CIT patients by different treatments obtained by Bayesian network meta-analysis were shown in Figure 6 and Supplementary Table S3. For grade 3/4 thrombocytopenia, rhTPO was most probable to rank lowest (SUCRA = 11.5), followed by eltrombopag (SUCRA = 37.8). For increasing nadir platelet count, eltrombopag ranked highest (SUCRA = 85.2). However, eltrombopag was most likely to rank lowest for chemotherapy dose reduction/delay (SUCRA = 11.1). Avatrombopag was superior in promoting neutrophil and hemoglobin recovery (SUCRA = 5.7 and 15.6, respectively). For AEs, avatrombopag exhibited the most favorable safety profile (SUCRA = 30.3), while rhIL-11 had the highest AEs rate (SUCRA = 89.5). Avatrombopag also showed the lowest thrombosis rate (SUCRA = 39.3). This suggested that avatrombopag may have the best safety profile.

#### 3.3.4 Heterogeneity and inconsistency assessment

Supplementary Figure S3 displays the forest plots along with the heterogeneity estimates for the eligible pairwise comparisons across the studies. Heterogeneity analysis was conducted using a Bayesian network meta-analysis framework. Over half of the comparisons for various outcomes demonstrated minimal (*I* ^2^ = 0%) or low to moderate (*I* ^2^ ≤ 50%) heterogeneity. Certain comparisons exhibited high heterogeneity (*I* ^2^ ≥ 50%), including placebo versus rhTPO in nadir platelet count (53.6%), and placebo versus eltrombopag and romiplostim in bleeding events (62.2% and 66.4%, respectively). The deviance information criterion, a Bayesian metric that accounts for both model fit and complexity, was used to compare the fit of consistent and inconsistent models. The consistency model in this network meta-analysis showed a similar or superior fit to the inconsistency model (Supplementary Table S2), suggesting favorable global consistency.

#### 3.3.5 Sensitivity analysis

A sensitivity analysis was performed to evaluate the reliability and robustness of the results in bleeding events by using a random-effects model. The results showed that eltrombopag ranked lowest for bleeding events, achieving a SUCRA value of 35.7, followed by romiplostim (47) and avatrombopag (49.7) (Supplementary Figure S5). Sensitivity analysis using a random-effects model showed that eltrombopag still had a significant advantage over placebo in terms of platelet nadir (SMD = −37.82, 95% CI: −62.93 to −12.71). Bayesian ranking probability analysis suggested that eltrombopag had the highest probability of ranking first in increasing the nadir platelet count (SUCRA = 80.8) (Supplementary Figure S5).

To address heterogeneity across studies (including chemotherapy regimens, tumor types, drug dose, and patient age), we performed sensitivity analyses using random-effects models through systematically excluding specific study types to evaluate the stability of core outcomes. When excluding single-agent chemotherapy studies (*n* = 4), the significant difference in adverse reactions between rhIL-11 and eltrombopag disappeared (OR = 2.40, 95% CI: 0.57–10.07 vs. the original OR = 2.97, 95% CI: 1.11–7.94), while other outcomes remained stable. Removal of hematologic malignancy studies (*n* = 3) only affected the eltrombopag-romiplostim comparison for platelet recovery ≥100 × 10^9^/L (OR changed from 0.02 to 0.05, 95% CI from 0.00 to 0.74 to 0.01–1.91). After excluding non-standard dose studies (*n* = 4), two changes emerged: First, eltrombopag no longer showed significant benefits for grade 3/4 thrombocytopenia (OR changed from 2.24 (1.13, 4.44) to 1.29 (0.42, 3.94)) and anemia (OR changed from 1.73 (1.04, 2.87) to 1.41 (0.68, 2.92)). Second, compared to placebo, avatrombopag (−23.40 (−54.71, 7.91) to −23.40 (−42.97, −3.83)), rhIL-11 (−18.45 (−42.27, 5.36) to −28.44 (−46.67, −10.22)), rhTPO (−18.80 (−40.96, 3.36) to −12.00 (−20.67, −3.33)) and mIL-11 (−20.71 (−56.63, 15.20) to −30.84 (−52.52, −9.17)) demonstrated significant improvements in nadir platelet count. All outcomes remained consistent when excluding the sole pediatric study. As shown in Supplementary Figures S6–S9, the sensitivity analyses demonstrated that most results were consistent with the primary findings, confirming the robustness of our study results.

## 4 Discussion

Traditional meta-analyses have primarily compared TPO-RAs, rhIL-11, and rhTPO with placebo in CIT treatment (Zhang et al., 2017; Liu et al., 2020; Soff et al., 2022). However, in addition to conducting traditional direct comparisons, our study also performed a network meta-analysis to evaluates six thrombopoietic agents. Direct meta-analysis revealed that TPO-RAs significantly reduced the incidence of grade 3/4 thrombocytopenia and increased platelet nadirs, emphasizing their role in preventing severe thrombocytopenia. After excluding a high heterogeneity study, TPO-RAs also reduced chemotherapy dose reductions and delays without increasing adverse event risk. The results of network meta-analysis demonstrated that eltrombopag had significant advantages in reducing grade 3/4 thrombocytopenia, improving platelet nadir, and preventing chemotherapy dose reductions or delays. The exclusion of three non-standard-dose eltrombopag studies rendered its benefits for grade 3/4 thrombocytopenia and anemia non-significant, suggesting these effects may require supra-therapeutic dosing for adequate megakaryocyte and hematopoietic stem cell stimulation. Avatrombopag, rhIL-11, rhTPO and mIL-11 showed newly significant platelet nadir improvements, likely because standard-dose agents’ effects were previously masked by eltrombopag’s potent dose-dependent apoptosis inhibition. In contrast, romiplostim only excelled in recovering platelet counts >100 × 10^9^/L, outperforming both eltrombopag and avatrombopag, likely due to eltrombopag’s oral administration and faster absorption (Profit, 2006), whereas romiplostim requires subcutaneous injection, exhibiting slower absorption and potentially affecting patient adherence (Bussel et al., 2021). *In vitro* studies suggest avatrombopag promotes megakaryocyte proliferation and differentiation more effectively than eltrombopag, and animal studies showed greater platelet count increases with avatrombopag (Abe et al., 2011; Xie et al., 2018). However, only one study comparing avatrombopag to placebo was included, with no direct comparisons to other agents, limiting accurate risk-benefit evaluations. Therefore, its efficacy and safety rankings should be interpreted with caution. In future, large-scale, rigorously designed, multicenter randomized controlled trials and head-to-head comparative studies between avatrombopag and other thrombopoietic agents are needed to validate the efficacy and safety of avatrombopag.

Our study revealed that rhTPO demonstrated a significantly faster onset of action in increasing platelet counts compared to rhIL-11 and TPO-RAs, likely due to its direct mimicry of endogenous TPO’s mechanism of action and its favorable pharmacokinetic profile (Kuter and Begley, 2002). Furthermore, the first comparative study between rhTPO and TPO-RAs indicated that rhTPO exhibits superior efficacy, making it the preferred option for emergency treatment (Mei et al., 2021). Although rhTPO has shown remarkable efficacy in mitigating grade 3/4 thrombocytopenia and accelerating platelet recovery to >100 × 10^9^/L (Mei et al., 2021; Yu et al., 2021), its similarity to endogenous TPO may lead to antibody development, potentially limiting its clinical use.

Studies have shown that IL-11 can accelerate platelet recovery after chemotherapy (Gordon et al., 1996; D'Hondt et al., 1995) and reduces platelet transfusion needs in CIT patients (Vredenburgh et al., 1998; Tepler et al., 1996), despite having limited effects on megakaryocyte proliferation and platelet production. Most studies on IL-11 have focused on unconventional chemotherapy regimens that cause severe thrombocytopenia. In contrast, studies on TPO-RAs and rhTPO have primarily involved standard chemotherapy regimens, where thrombocytopenia is less common. Consequently, the overall impact of TPO on platelet transfusion requirements may be limited (Kuter, 2002). This discrepancy may explain why our analysis found that rhIL-11 and mIL-11 significantly reduced platelet transfusion needs, while other agents showed no significant differences. Despite reducing platelet transfusion requirements by approximately one-third, IL-11 is associated with various adverse effects (Gordon et al., 1996; Vredenburgh et al., 1998). Our analysis also revealed that IL-11 had the highest incidence of adverse effects among the treatments studied, making it less favorable in terms of safety.

Studies have reported that TPO-RAs promote trilineage hematopoietic recovery in patients with severe aplastic anemia (Gilreath et al., 2021). The c-MPL receptor is broadly expressed across hematopoietic lineages, including CD34^+^ stem cells, multipotent progenitors, erythroid precursors, and granulocyte-macrophage progenitors (Plo et al., 2017). TPO-RAs may affect multiple hematopoietic lineages beyond the megakaryocyte lineage by enhancing the proliferation and differentiation of progenitor cells, offering potential therapeutic benefits for patients with various cytopenias (Plo et al., 2017). Beyond their established role in thrombopoiesis, TPO-RAs exert multilineage effects by activating JAK2-STAT5/BCL-xL survival pathways while simultaneously stimulating PI3K-AKT-mTOR mediated proliferation (Kaushansky et al., 1996). These agents further modulate the bone marrow microenvironment through CXCL12 upregulation (Yoshihara et al., 2007) to enhance stem cell homing and by counteracting IFN-γ-mediated inflammatory suppression of hematopoiesis (Alvarado et al., 2019). TPO plays a critical role in hematopoietic maintenance and stem cell niche regulation (de Graaf and Metcalf, 2011). Our network meta-analysis found that TPO-RAs significantly reduced the incidence of anemia and neutropenia, indicating that they not only stimulate platelet production but also potentially promote the recovery of neutrophils and hemoglobin.

Cancer patients undergoing chemotherapy are inherently at higher risk of thrombosis (Fernandes et al., 2019), and increased platelet count could further elevate the risk of thromboembolic events. TPO-RAs have been linked to an increased risk of thrombosis, though the mechanisms remain unclear (Guitton et al., 2018). Thrombotic events were reported with eltrombopag and romiplostim (Kuter et al., 2008). However, no significant difference in thrombosis rates was found between the treatment and placebo groups, suggesting TPO-RAs do not notably increase thrombosis risk in CIT patients.

Although mechanistic studies suggest that TPO-RAs may theoretically increase thrombotic risk through platelet activation, clinical data from randomized controlled trials and meta-analyses have not demonstrated a statistically significant increase in thromboembolic events (Murphy, 2022; Wong et al., 2017). This apparent paradox may be attributed to the combined effects of: low baseline platelet counts limit platelet activation, preventing thrombosis risk threshold from being reached (Khorana et al., 2008), trial designs that excluded high-risk patients, TPO-RAs dosing strategies aimed at maintaining hemostatic (rather than normal) platelet levels, and chemotherapy-induced myelosuppression creating a protective threshold effect (Grozovsky et al., 2015). Similar to observations with erythropoietin (Henke et al., 2003), these findings illustrate how preclinical thrombotic potential may not necessarily translate into clinical risks in thrombocytopenic populations. Further studies evaluating long-term risks in real-world settings are warranted.

Different chemotherapeutic drugs such as alkylating agents and cyclophosphamide have distinct regulatory pathways in megakaryocyte development (Song and Al-Samkari, 2023), which may affect the therapeutic response to thrombopoietic agents. Non-CYP metabolized TPO-RAs including avatrombopag and romiplostim show lower risks of drug interactions with common chemotherapeutic agents (e.g., paclitaxel, irinotecan) due to their metabolic characteristics (Kuter, 2022; Katsube et al., 2020). However, caution is needed when using the CYP-metabolized agent eltrombopag in combination with certain chemotherapy drugs. Special attention should be paid to the potential increased risk of DNA damage in hematopoietic stem cells when eltrombopag is co-administered with topoisomerase inhibitors due to its iron-chelating effects (Roth et al., 2012). Therefore, we recommend a sequential administration strategy, such as initiating TPO-RAs treatment after an interval following chemotherapy. Future prospective studies incorporating PK/PD modeling and *in vitro* screening are needed to further elucidate the interaction mechanisms between chemotherapeutic agents and thrombopoietic agents.

However, our study has several limitations. First, differences in study designs across the included trials led to incomplete data extraction. Due to the limited number of studies, subgroup analyses based on different drug dose and patient characteristics could not be performed. Secondly, only a few endpoints they interested in were assessed in the evaluated studies. As a result, data for specific endpoints were drawn from a limited number of studies, and outcome definitions varied among studies. Thirdly, some drugs involved few articles, and results must be interpreted with caution when compared with other drugs. Finally, the efficacy and cost burden of certain drugs could not be evaluated due to the lack of original data and cost-effectiveness analyses. These uncertainties and debates can only be resolved by acquiring more reliable data through future trials, such as additional RCTs or head-to-head studies incorporating long-term follow-up data and economic benefit evaluation.

The study demonstrates that eltrombopag outperforms romiplostim and avatrombopag in the evaluated therapeutic efficacy indicators. Regarding adverse effects, avatrombopag is associated with the fewest reactions. But these findings require further confirmation in larger, well-controlled trials. rhTPO effectively raises platelet counts but carries the risk of inducing endogenous TPO antibody production. rhIL-11 significantly reduces platelet transfusions, yet its use is limited by notable adverse effects. In conclusion, although TPO-RAs, particularly eltrombopag, appear optimal for treating CIT, clinical decisions should consider individual patient needs, drug properties, and preferences.

## Data Availability

The original contributions presented in the study are included in the article/Supplementary Material, further inquiries can be directed to the corresponding author.
