# Supplementary material for: Efficacy and safety of different treatments in chemotherapy-induced thrombocytopenia: a systematic review and network meta-analysis
Source: Front Pharmacol. 2025 Jul 22;16:1549214. doi: 10.3389/fphar.2025.1549214 (PMC12321752; doi:10.3389/fphar.2025.1549214)
Supplement: Supplementary file 1 [file DataSheet1.pdf]

Supplementary materials

Table of Contents

		Page
**Supplementary Table 1**	Literature search criteria	**2-5**
**Supplementary Table 2**	Comparisons of the fit of consistency and inconsistency	**5-5**
**Supplementary Table 3**	Bayesian ranking results of network meta-analysis	**6-7**
**Supplementary Figure 1**	Convergence of the three chains established by trace and the Brooks-Gelman-Rubin diagnostic	**7-14**
**Supplementary Figure 2**	Results from assessment of studies using the Cochrane risk of bias tool.	**14-15**
**Supplementary Figure 3**	Forest plots depicting results of head-to-head comparisons according to Bayesian pairwise and network meta-Analyses.	**15-22**
**Supplementary Figure4**	Comparison-adjusted funnel plot	**22-27**
**Supplementary Figure5**	Sensitive analysis of network meta-analysis on bleeding events and nadir platelet count	**27-29**
**Supplementary Figure6**	Sensitive analysis of network meta-analysis with the exclusion of a pediatric study.	**30**
**Supplementary Figure7**	Sensitive analysis of network meta-analysis after excluding the three studies on hematologic malignancies.	**31-33**
**Supplementary Figure8**	Sensitive analysis of network meta-analysis after excluding the four studies on single-agent chemotherapy.	**35-36**

**Supplementary Figure9**	Sensitive analysis of network meta-analysis after excluding the four studies on non-standard doses.	**35-39**

Supplementary Table 1. Literature search criteria.

Database	RCTs	Literature search criteria
**PubMed**	158	((((((((((chemotherapy-induced thrombocytopenia[Title/Abstract]) OR (cancer therapy-related thrombocytopenia[Title/Abstract])) OR (thrombocytopenia[Title/Abstract])) OR (macrothrombocytopenia[Title/Abstract])) OR (platelet count decreased[Title/Abstract])) OR (platelet deficiency[Title/Abstract])) OR (thrombocyte deficiency[Title/Abstract])) OR (thrombocytopaenias[Title/Abstract])) OR (thrombopenia[Title/Abstract])) OR (thrombopenias[Title/Abstract])) AND ((((((((((((((((((((romiplostim[Title/Abstract]) OR (AMG 531[Title/Abstract])) OR (Nplate[Title/Abstract])) OR (Romiplate[Title/Abstract])) OR (eltrombopag[Title/Abstract])) OR (Promacta[Title/Abstract])) OR (Revolade[Title/Abstract])) OR (thrombopoietin[Title/Abstract])) OR (TPO[Title/Abstract])) OR (TPO-Ras[Title/Abstract])) OR (thrombopoietin receptor agonists[Title/Abstract])) OR (thrombopoietin mimetics[Title/Abstract])) OR (thrombopoietin stimulating agent[Title/Abstract])) OR (herombopag[Title/Abstract])) OR (avatrombopag[Title/Abstract])) OR (lusutrombopag[Title/Abstract])) OR (rhIL-11[Title/Abstract])) OR (Recombinant human interleukin-11[Title/Abstract])) OR (Oprelvekin[Title/Abstract])) OR (Neumega[Title/Abstract])) OR (platelet transfusion[Title/Abstract])) OR (recombinant human thrombopoietin[Title/Abstract])) OR (rhTPO[Title/Abstract])))Filters: Randomized Controlled Trial

**cochrane**	341	#1 (romiplostim): ti,ab,kw OR (AMG 531):ti,ab,kw OR (Nplate):ti,ab,kw OR (Romiplate):ti,ab,kw OR (eltrombopag):ti,ab,kw<br><br>#2 (Promacta):ti,ab,kw OR (Revolade):ti,ab,kw OR (thrombopoietin):ti,ab,kw OR (TPO):ti,ab,kw OR (TPO-Ras):ti,ab,kw<br><br>#3 (thrombopoietin receptor agonists): ti,ab,kw OR (thrombopoietin mimetics):ti,ab,kw OR (thrombopoietin stimulating agent):ti,ab,kw OR (herombopag):ti,ab,kw OR (avatrombopag):ti,ab,kw<br>#4 (lusutrombopag): ti,ab,kw OR (rhIL-11):ti,ab,kw OR (Recombinant human interleukin-11):ti,ab,kw OR (Oprelvekin):ti,ab,kw OR (Neumega):ti,ab,kw<br>#5 (recombinant human thrombopoietin): ti,ab,kw OR (rhTPO):ti,ab,kw<br>#6 (Randomized Controlled Trial): ti,ab,kw 776494<br>#7 (chemotherapy-induced thrombocytopenia): ti,ab,kw OR (cancer therapy-related thrombocytopenia):ti,ab,kw OR (thrombocytopenia):ti,ab,kw OR (macrothrombocytopaenia):ti,ab,kw OR (platelet count decreased):ti,ab,kw<br>#8 (platelet deficiency): ti,ab,kw OR (thrombocyte deficiency):ti,ab,kw OR (thrombocytopaenias):ti,ab,kw OR (thrombopenia):ti,ab,kw OR (thrombopenias):ti,ab,kw<br>#9 #1 OR #2 OR #3 OR #4 OR #5<br>#10 #7 OR #8<br>#11 #6 AND #9 AND #10
**Embase**	387	#1：romiplostim:ab,ti OR 'amg 531':ab,ti OR nplate:ab,ti OR romiplate:ab,ti OR eltrombopag:ab,ti OR promacta:ab,ti OR revolade:ab,ti OR thrombopoietin:ab,ti OR tpo:ab,ti OR 'tpo ras':ab,ti OR 'thrombopoietin receptor agonists':ab,ti OR 'thrombopoietin mimetics':ab,ti OR 'thrombopoietin stimulating agent':ab,ti OR herombopag:ab,ti OR avatrombopag:ab,ti OR lusutrombopag:ab,ti OR rhil-11:ab,ti OR 'recombinant human interleukin-11':ab,ti OR oprelvekin:ab,ti OR neumega:ab,ti OR 'platelet transfusion':ab,ti OR 'recombinant human thrombopoietin':ab,ti OR rhtpo:ab,ti<br>#2：'chemotherapy-induced thrombocytopenia': ab,ti OR 'cancer therapy-related thrombocytopenia':ab,tiOR thrombocytopenia:ab,ti OR macrothrombocytopaenia:ab,ti OR 'platelet count decreased':ab,ti OR 'platelet deficiency':ab,ti OR 'thrombocyte deficiency':ab,ti OR thrombocytopaenias:ab,ti OR thrombopenia:ab,ti OR thrombopenias:ab,ti<br>#3：#1 AND #2 Filters: Randomized Controlled Trial

ClinicalTrials.gov	13	Intervention/treatment： romiplostim OR 'amg 531'<br>OR nplateOR romiplateOR eltrombopagOR promactaOR revolade<br>OR thrombopoietin OR tpo OR 'tpo ras' OR 'thrombopoietin receptor agonists' OR 'thrombopoietin mimetics' OR 'thrombopoietin stimulating agent' OR herombopag OR avatrombopag OR lusutrombopag OR rhil-11<br>OR 'recombinant human interleukin-11' OR oprelvekin OR neumega<br>OR 'platelet transfusion' OR 'recombinant human thrombopoietin'<br>OR rhtpo<br>Condition/disease： chemotherapy-induced thrombocytopenia
web of science	233	#1:((((((((((((((((((((AB=(romiplostim)) OR AB=(AMG 531)) OR AB=(Nplate)) OR AB=(Romiplate)) OR AB=(eltrombopag)) OR AB=(Promacta)) OR AB=(Revolade)) OR AB=(thrombopoietin)) OR AB=(TPO)) OR AB=(TPO-Ras)) OR AB=(thrombopoietin receptor agonists)) OR AB=(thrombopoietin mimetics)) OR<br>AB=(thrombopoietin stimulating agent)) OR AB=(herombopag)) OR AB=(avatrombopag)) OR AB=(lusutrombopag)) OR AB=(rhIL-11)) OR AB=(Recombinant human interleukin-11)) OR AB=(Oprelvekin)) OR AB=(Neumega)) OR AB=(platelet transfusion)) OR AB=(recombinant human thrombopoietin)) OR AB=(rhTPO)<br>#2：((((((((AB= (chemotherapy-induced thrombocytopenia)) OR AB= (cancer therapy-related thrombocytopenia)) OR<br>AB=(thrombocytopenia)) OR AB= (macrothrombocytopaenia)) OR AB=(platelet count decreased)) OR AB=(platelet deficiency)) OR AB=(thrombocyte deficiency)) OR AB=(thrombocytopaenias)) OR AB=(thrombopenia)) OR AB=(thrombopenias)<br>#3：AB= (randomized controlled trial)<br>#1 AND #2 AND #3<br><br>#1：((((((((((((((((((((TI=(romiplostim)) OR TI=(AMG 531)) OR TI=(Nplate)) OR TI=(Romiplate)) OR TI=(eltrombopag)) OR TI=(Promacta)) OR TI=(Revolade)) OR TI=(thrombopoietin)) OR TI=(TPO)) OR TI=(TPO-Ras)) OR TI=(thrombopoietin receptor agonists)) OR TI=(thrombopoietin mimetics)) OR TI=(thrombopoietin stimulating agent)) OR TI=(herombopag)) OR TI=(avatrombopag)) OR TI=(lusutrombopag)) OR TI=(rhIL-11)) OR TI=(Recombinant human interleukin-11)) OR TI=(Oprelvekin)) OR TI=(Neumega)) OR TI=(platelet transfusion)) OR TI=(recombinant human thrombopoietin)) OR TI=(rhTPO)<br>#2：((((((((TI= (chemotherapy-induced thrombocytopenia)) OR TI= (cancer therapy-related thrombocytopenia)) OR TI=(thrombocytopenia)) OR TI= (macrothrombocytopaenia)) OR TI= (platelet count decreased))

		OR TI= (platelet deficiency)) OR TI= (thrombocyte deficiency)) OR TI=(thrombocytopaenias)) OR TI=(thrombopenia)) OR TI=(thrombopenias)<br>#3：TI= (randomized controlled trial)<br>#1 AND #2 AND #3

Supplementary Table 2. Comparisons of the fit of consistency and inconsistency.

	Overall		
Model	Adverse events	Platelet count recovery to ≥100×10^9	Platelet count recovery to ≥100×10^9 (d)
Consistency	45.98	15.00	15.00
Inconsistency	45.79	16.02	16.02

	Overall		
Model	Grade 3/4 thrombocytopenia	Bleeding events	Delays/Dose reductions/missed doses due to thrombocytopenia
Consistency	22.79	22.01	15.50
Inconsistency	22.59	22.01	15.66

	Overall		
Model	Neutropenia	Thromboembolic events	Anemia
Consistency	16.25	22.18	16.38
Inconsistency	16.29	22.19	16.35

	Overall	
Model	Nadir platelet count	Platelet transfusion
Consistency	27.97	40.99
Inconsistency	27.84	40.88

Supplementary Table 3. Bayesian ranking results of network meta-analysis.

Treatment	SUCRA	MeanRank
Placebo	83.7	2.0
Eltrombopag	64.9	3.1
Avatrombopag	86.9	1.8
rhIL-11	30.8	5.1
rhTPO	20.3	5.8
mIL-11	12.3	6.3
Romiplostim	51.0	3.9

A. Platelet transfusion

Treatment	SUCRA	MeanRank
Placebo	87.4	1.5
Eltrombopag	37.8	3.5
rhIL-11	39.9	3.4
rhTPO	11.5	4.5
Romiplostim	73.4	2.1

B. Grade 3/4 thrombocytopenia

Treatment	SUCRA	MeanRank
Placebo	22.0	3.3
Eltrombopag	39.7	2.8
Avatrombopag	39.5	2.8
Romiplostim	98.8	1.0

B. Platelet count recovery to ≥100×10^{9}/L

Treatment	SUCRA	MeanRank
Placebo	87.5	1.4
Eltrombopag	74.2	1.8
rhIL-11	34.6	3.0
rhTPO	3.7	3.9

D. Platelet count recovery to ≥100×10^{9}/L(d)

Treatment	SUCRA	MeanRank
Placebo	6.6	5.7
Eltrombopag	85.2	1.7
Avatrombopag	58.8	3.1
rhIL-11	47.6	3.6
rhTPO	49.2	3.5
mIL-11	52.6	3.4

E. Nadir platelet count

Treatment	SUCRA	MeanRank
Placebo	57.7	2.3
Eltrombopag	11.1	3.7
Avatrombopag	78.5	1.6
Romiplostim	52.7	2.4

F. Delays/Dose reductions/missed doses due thrombocytopenia

Treatment	SUCRA	MeanRank
Placebo	61.2	2.0
Eltrombopag	29.8	2.9
Avatrombopag	47.8	2.5
Romiplostim	61.1	2.6

G. Bleeding events

Treatment	SUCRA	MeanRank
Placebo	95.7	1.1
Eltrombopag	38.7	2.2
Avatrombopag	15.6	2.7

H. Anemia

Treatment	SUCRA	MeanRank
Placebo	99.0	1.0
Eltrombopag	45.3	2.1
Avatrombopag	5.7	2.9

I. Neutropenia

Treatment	SUCRA	MeanRank
Placebo	31.0	3.1
Eltrombopag	59.0	2.2
Avatrombopag	39.3	2.8
Romiplostim	70.6	1.9

J. Thromboembolic events

Treatment	SUCRA	MeanRank
Placebo	17.5	5.9
Eltrombopag	40.7	4.6
Avatrombopag	30.3	5.2
rhIL-11	89.5	1.6
rhTPO	74.8	2.5
mIL-11	66.1	3.0
Romiplostim	31.1	5.1

K. Adverse events

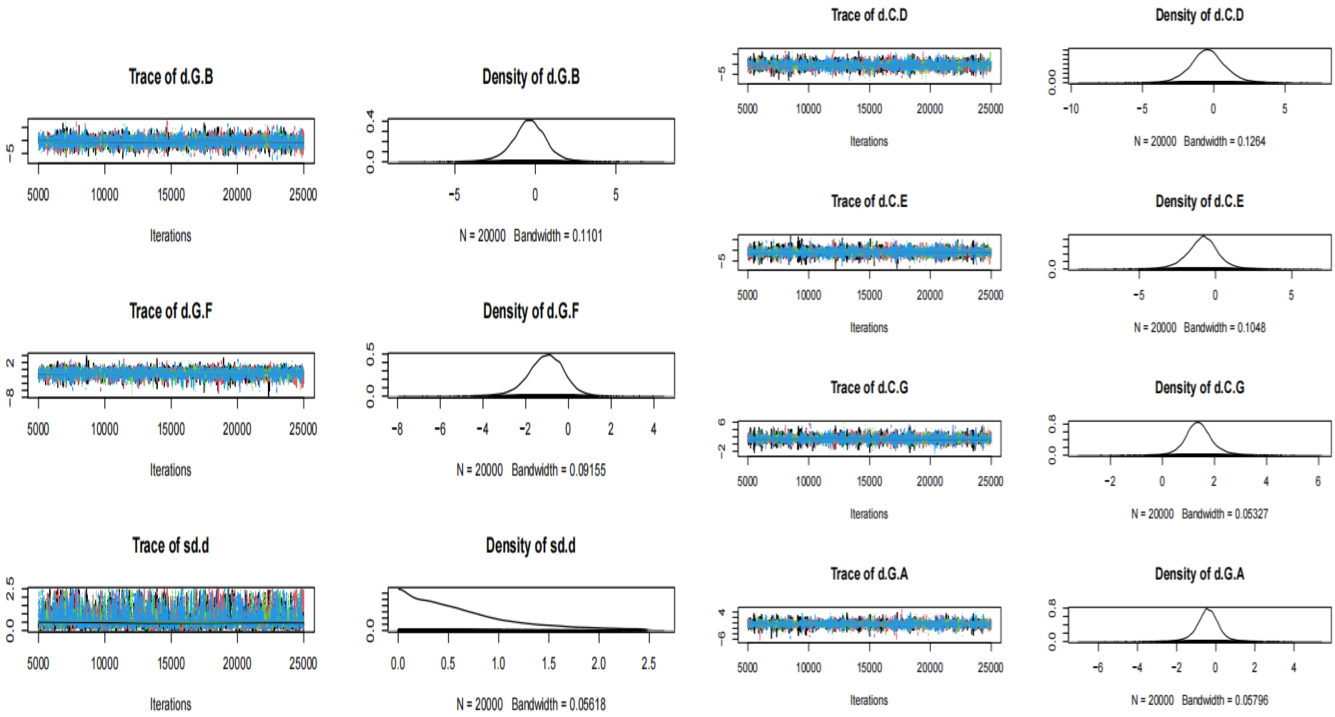

A. Trace for platelet transfusion

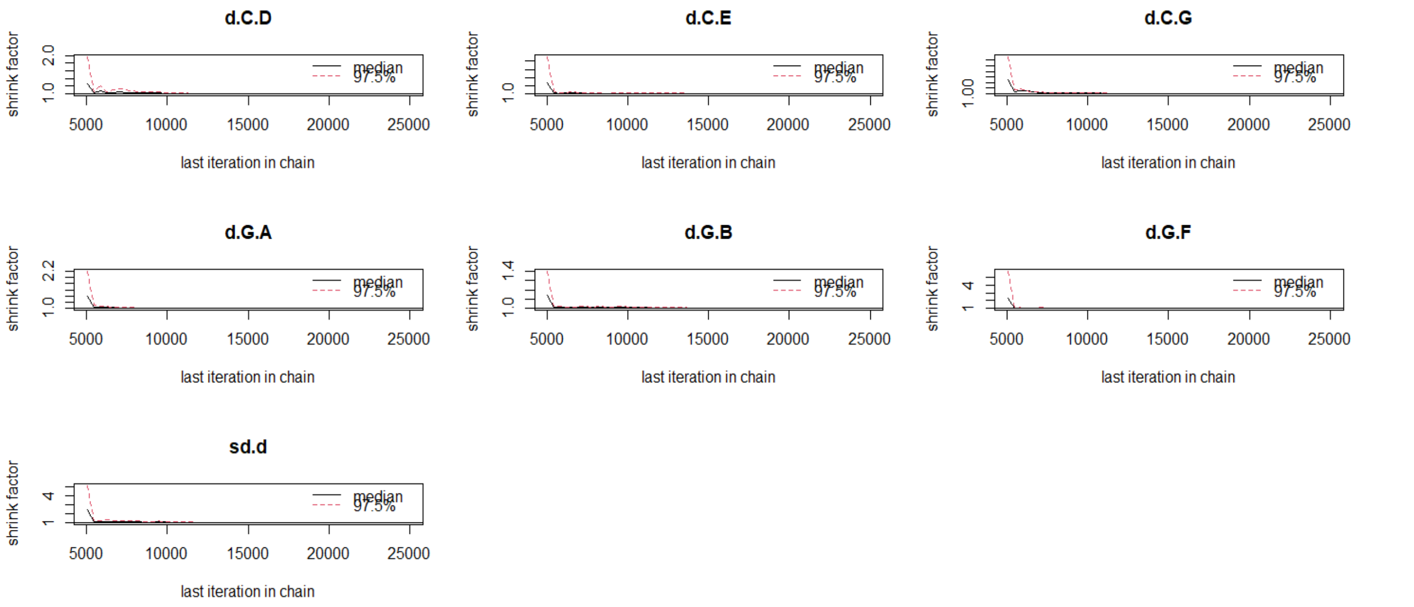

B. Brooks-Gelman-Rubin diagnostic for platelet transfusion

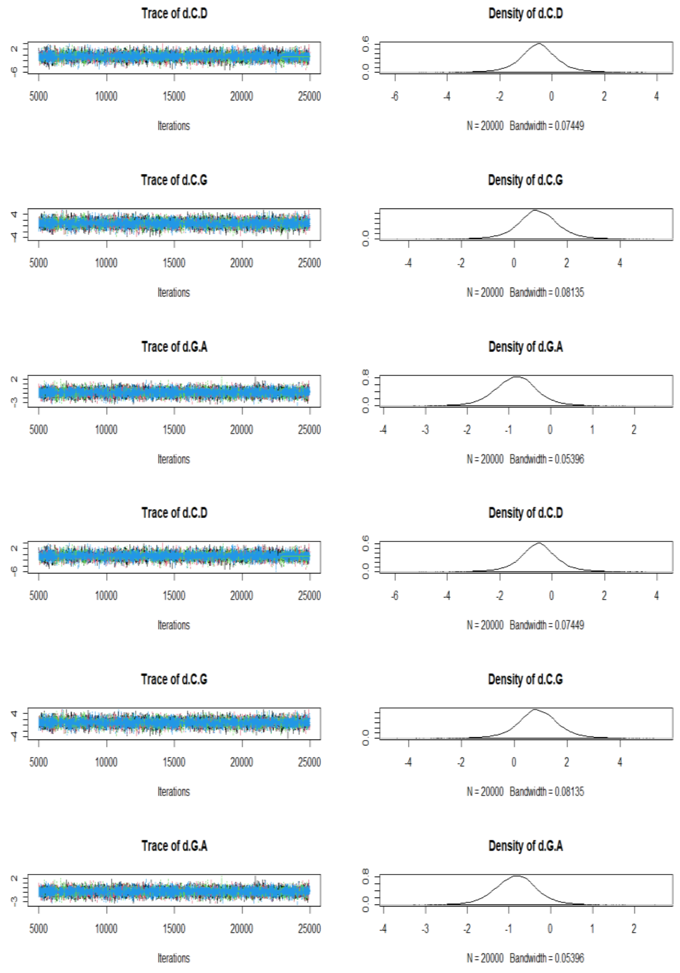

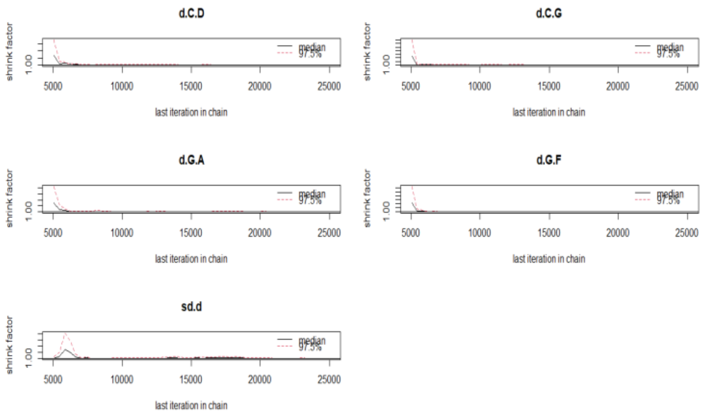

C. Trace for grade 3/4 thrombocytopenia

D. Brooks-Gelman-Rubin diagnostic for grade 3/4 thrombocytopenia

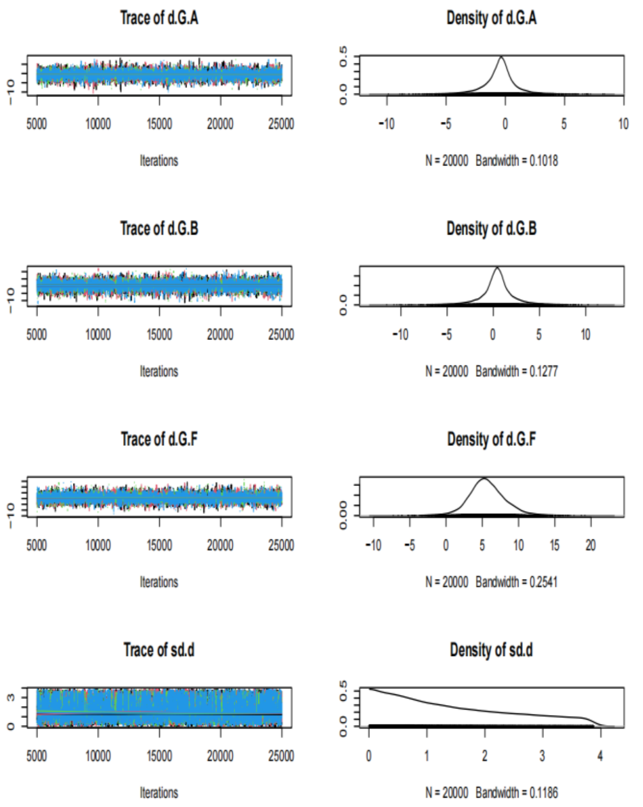

E. Trace for platelet count recovery to $\geq 100\times 10^9$/L

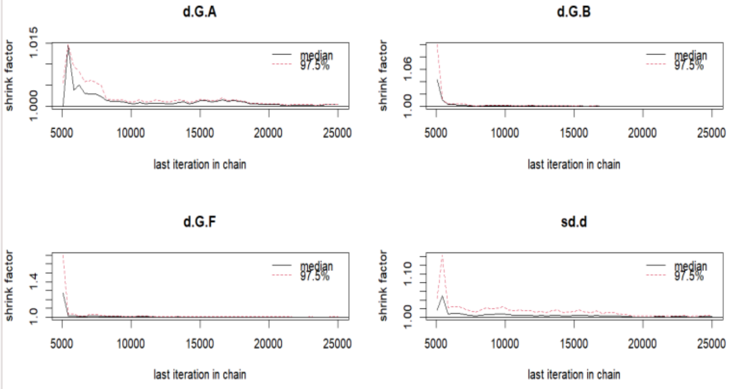

F. Brooks-Gelman-Rubin diagnostic for platelet count recovery to $\geq 100\times 10^9$/L

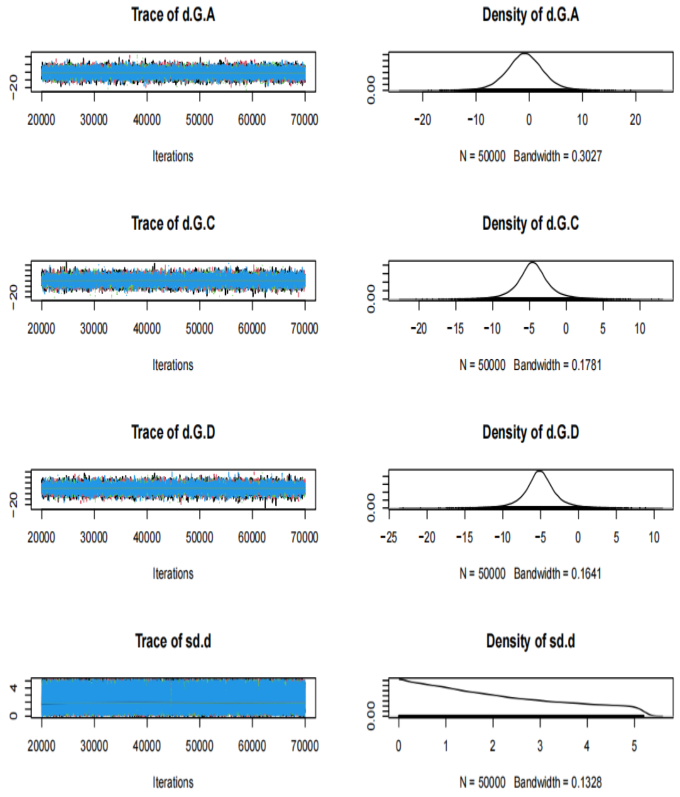

G. Trace for platelet count recovery to $\geq 100\times 10^9$(d)

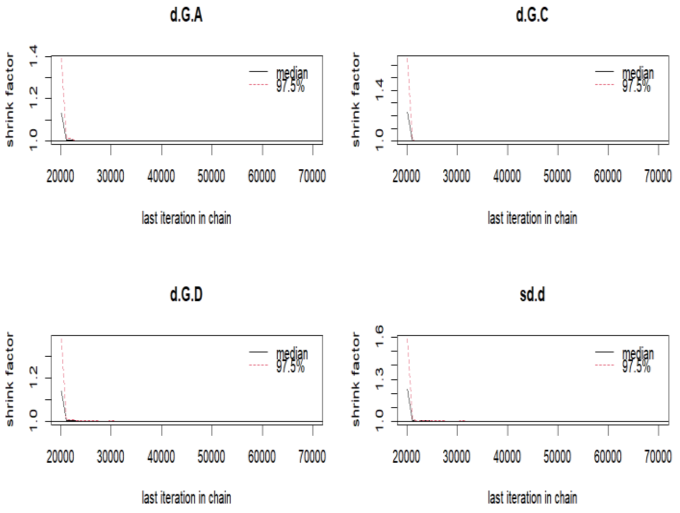

H. Brooks-Gelman-Rubin diagnostic for Platelet count recovery to $\geq 100\times 10^9$(d)

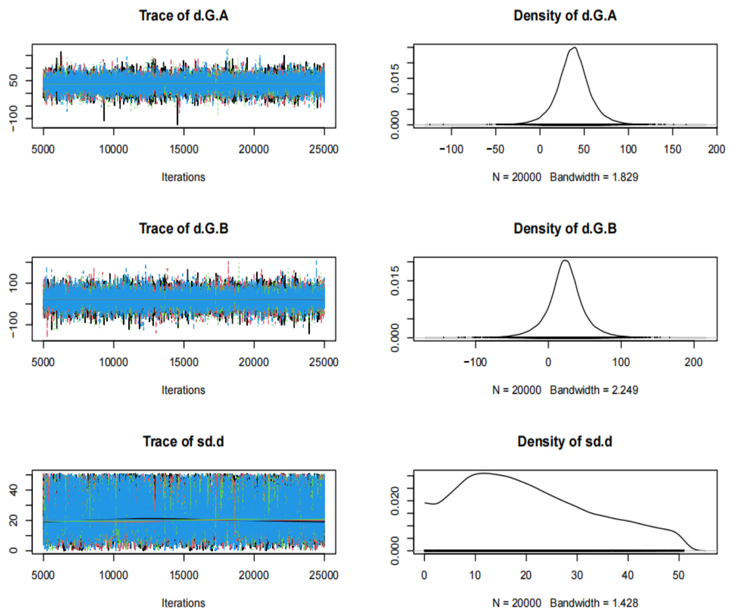

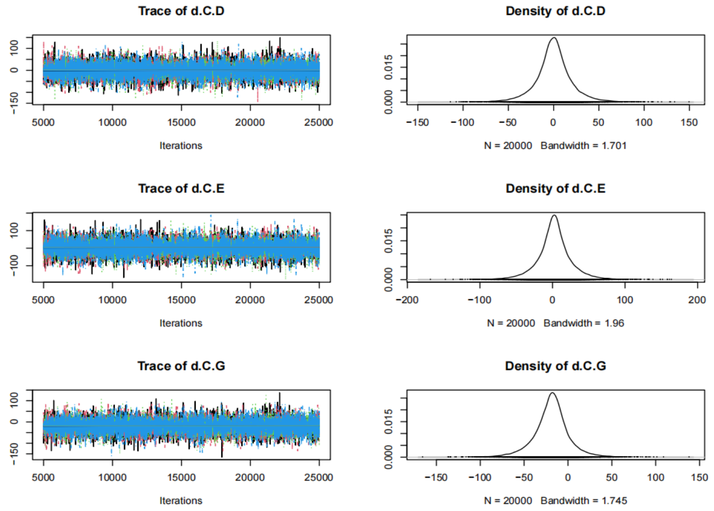

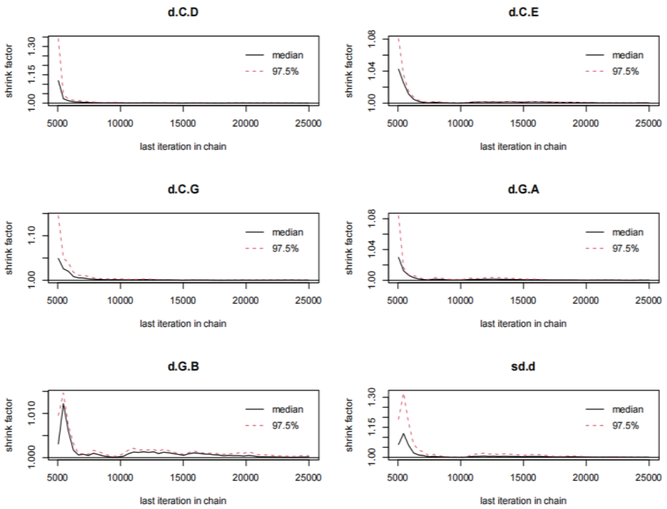

I. Trace for nadir platelet count

J. Brooks-Gelman-Rubin diagnostic for nadir platelet count

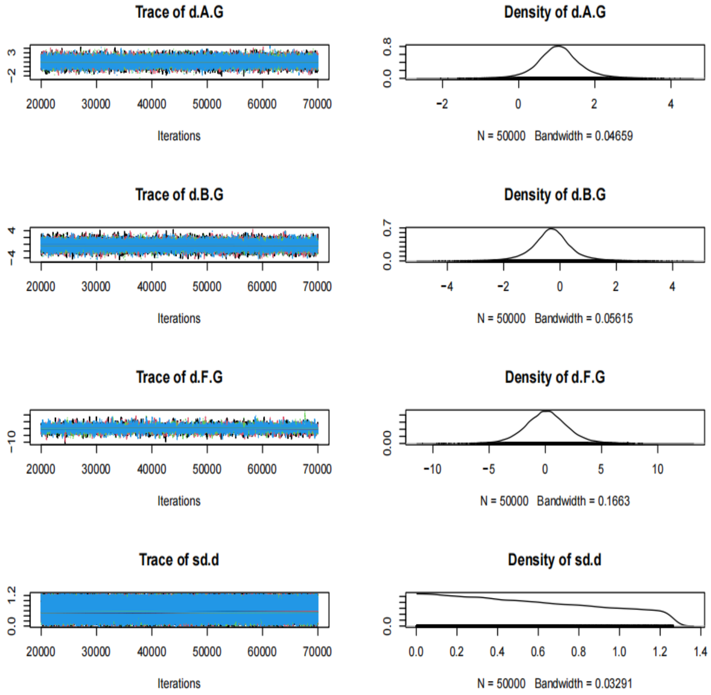

K. Trace for delays/dose reductions due to thrombocytopenia

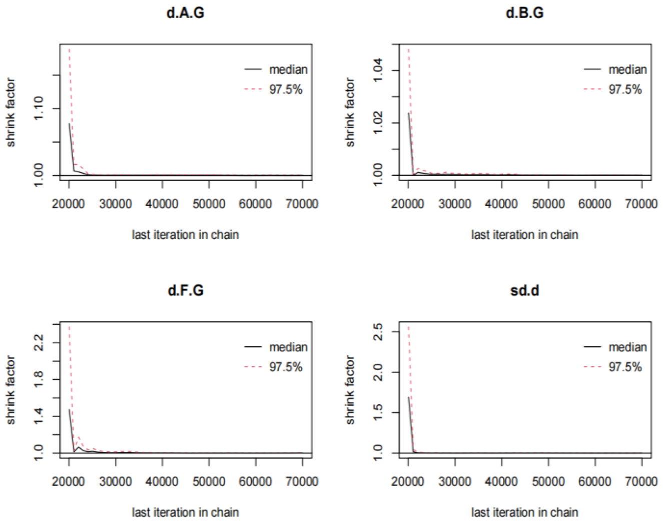

L. Brooks-Gelman-Rubin diagnostic for delays/dose reductions due to thrombocytopenia

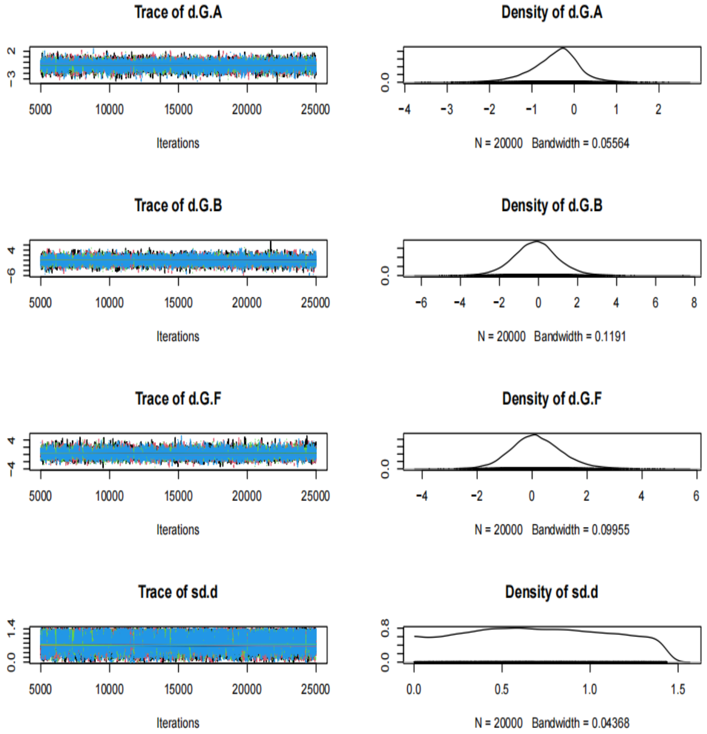

M. Trace for bleeding events

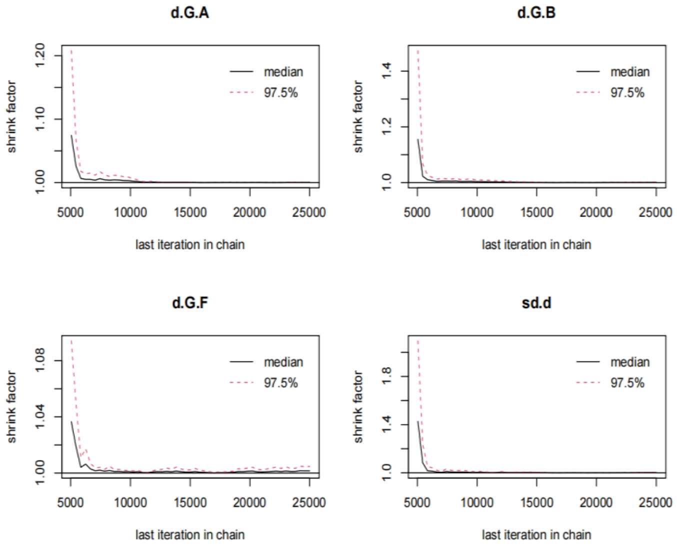

N. Brooks-Gelman-Rubin diagnostic for bleeding events

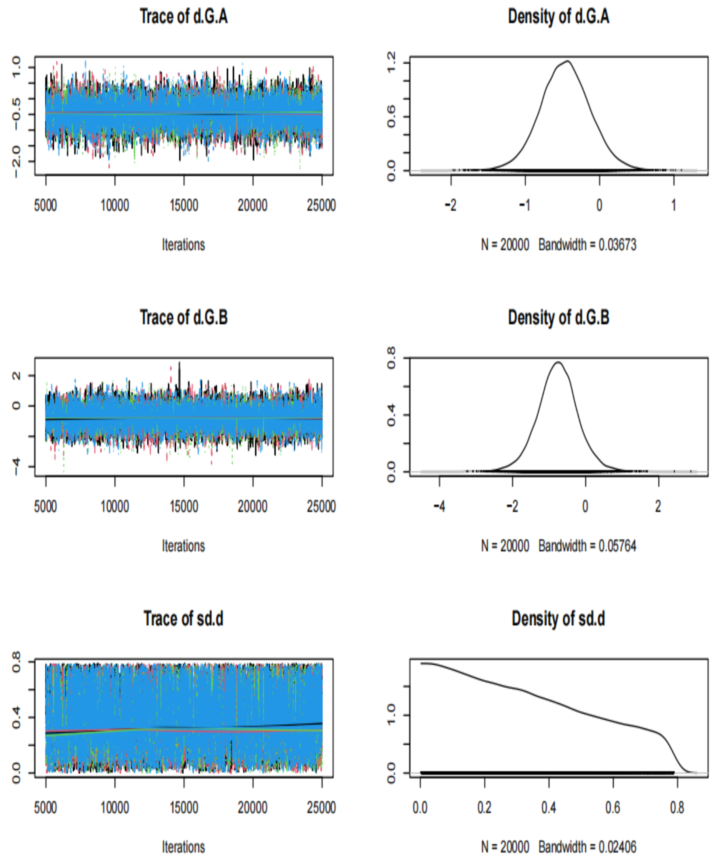

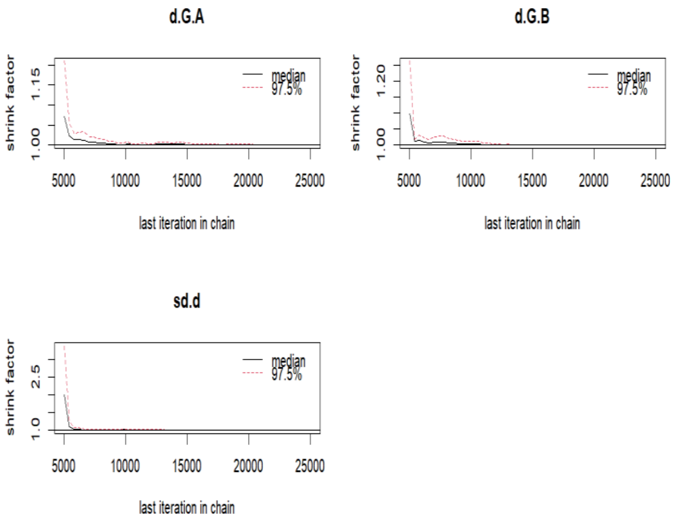

O. Trace for anemia

P. Brooks-Gelman-Rubin diagnostic for anemia

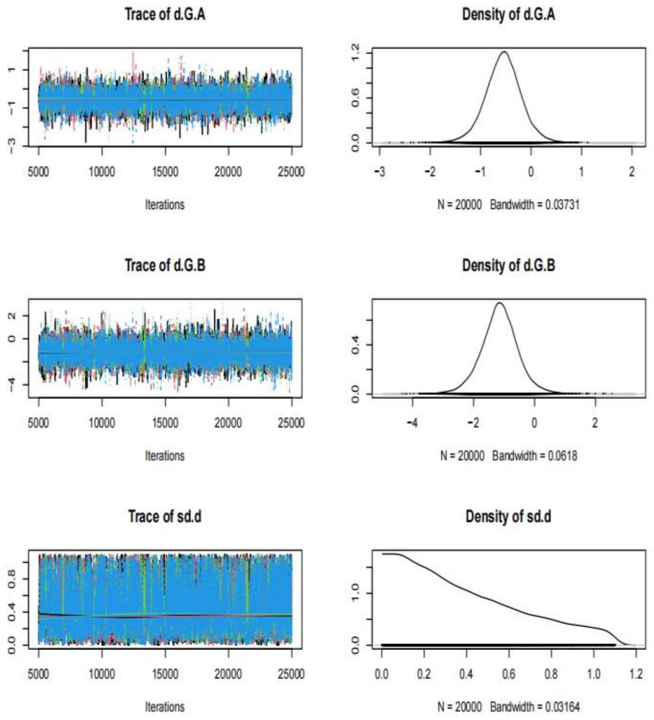

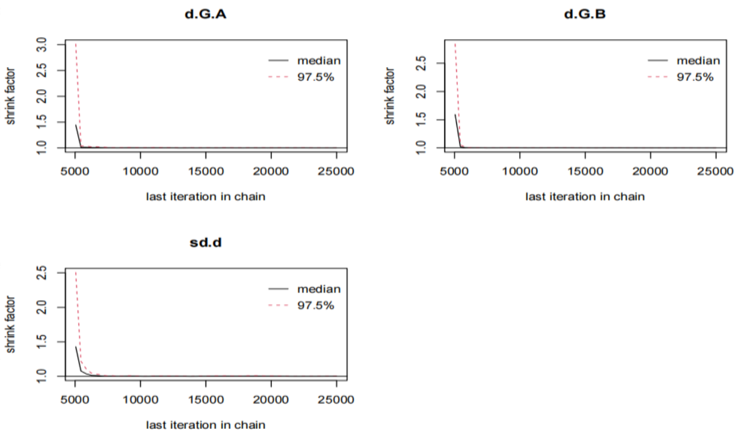

Q. Trace for neutropenia

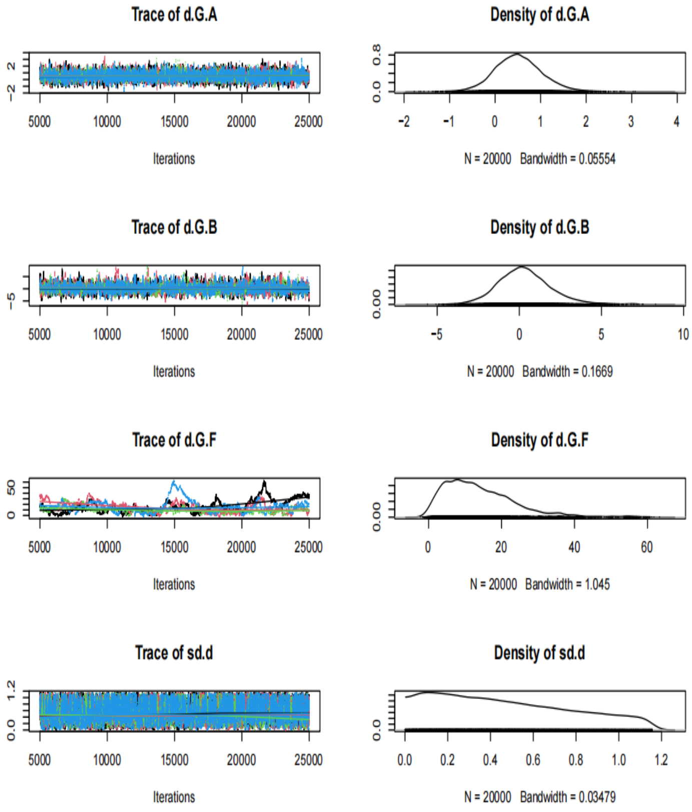

R. Brooks-Gelman-Rubin diagnostic for neutropenia

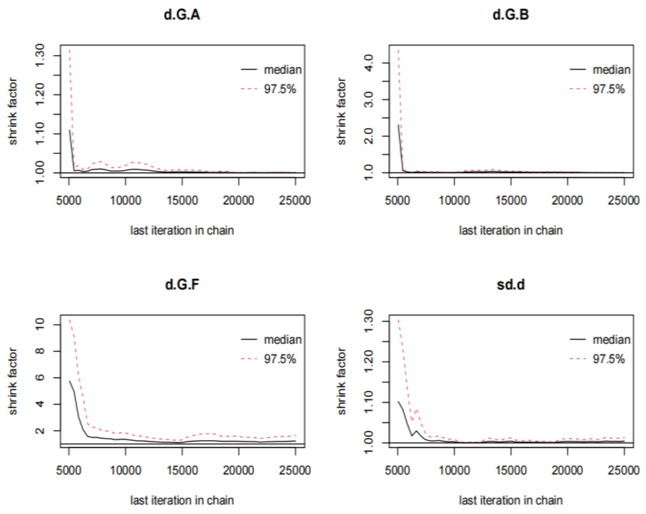

S. Trace for thromboembolic events

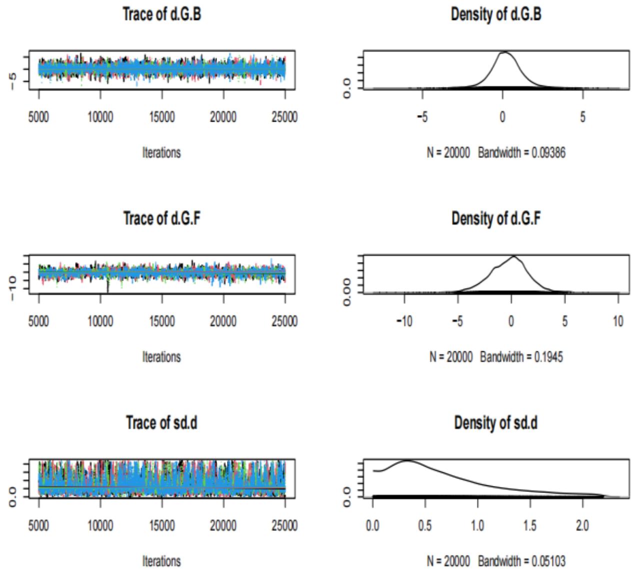

T. Brooks-Gelman-Rubin diagnostic for thromboembolic events

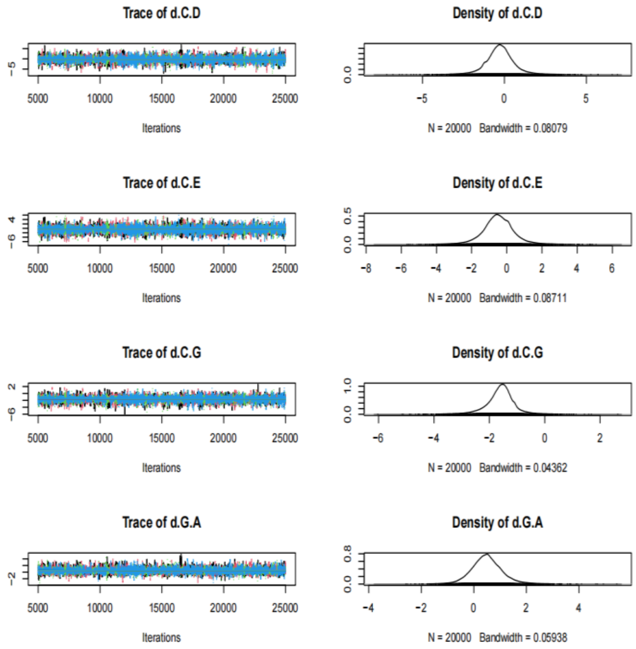

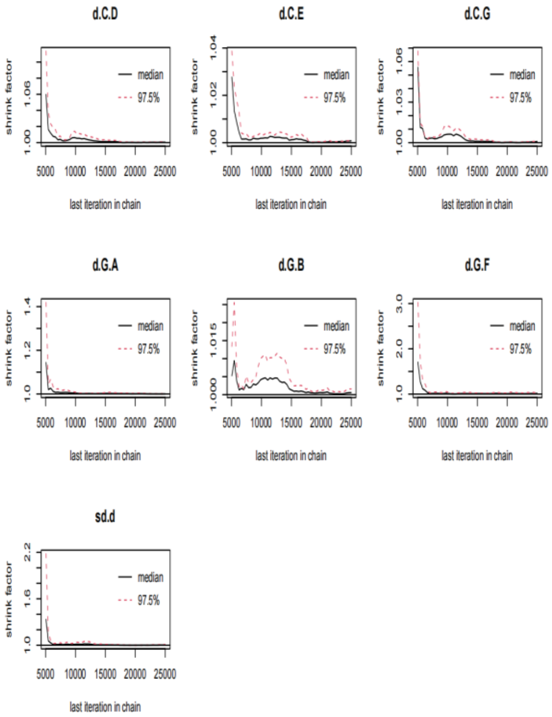

U. Trace for adverse events

V. Brooks-Gelman-Rubin diagnostic for adverse events

Supplementary Figure 1. Convergence of the three chains established by trace and the Brooks-Gelman-Rubin diagnostic for platelet transfusion (A and B), grade 3/4 thrombocytopenia (C and D), platelet count recovery to ≥100×10^9 (E and F), platelet count recovery to ≥100×10^9(d) (G and H), nadir platelet count (I and J), delays/dose reductions due to thrombocytopenia (K and L), bleeding events (M and N), anemia (O and P), neutropenia (Q and R), thromboembolic events (S and T), adverse events (U and V):

A. Risk of bias graph

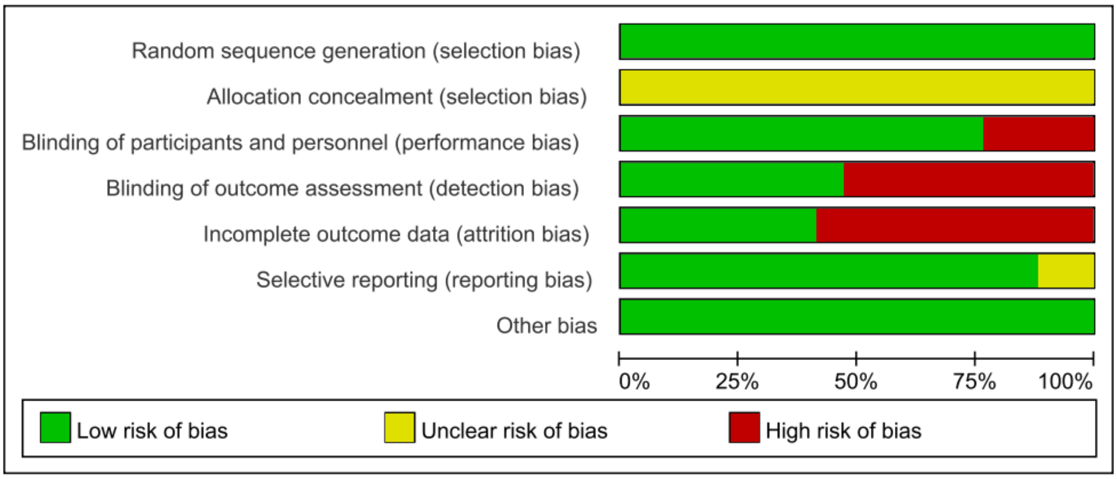

B. Risk of bias summary

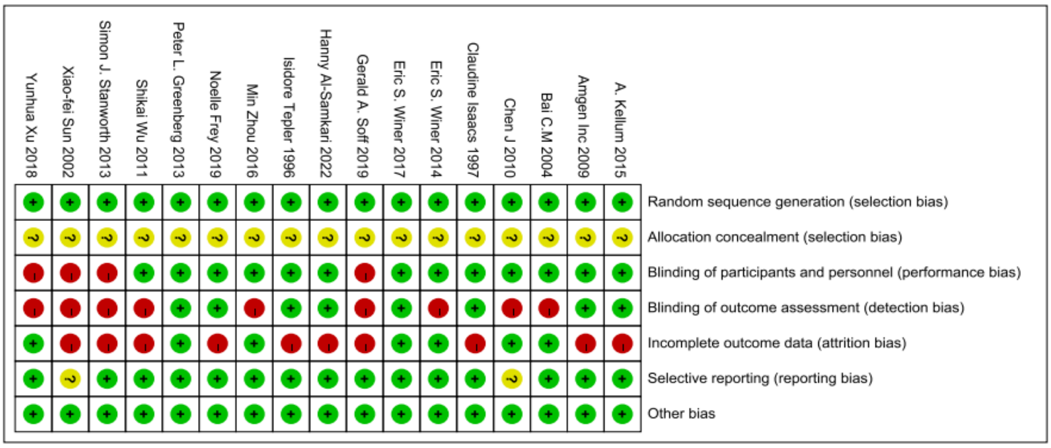

Supplementary Figure 2. Results from assessment of studies using the Cochrane risk of bias tool.

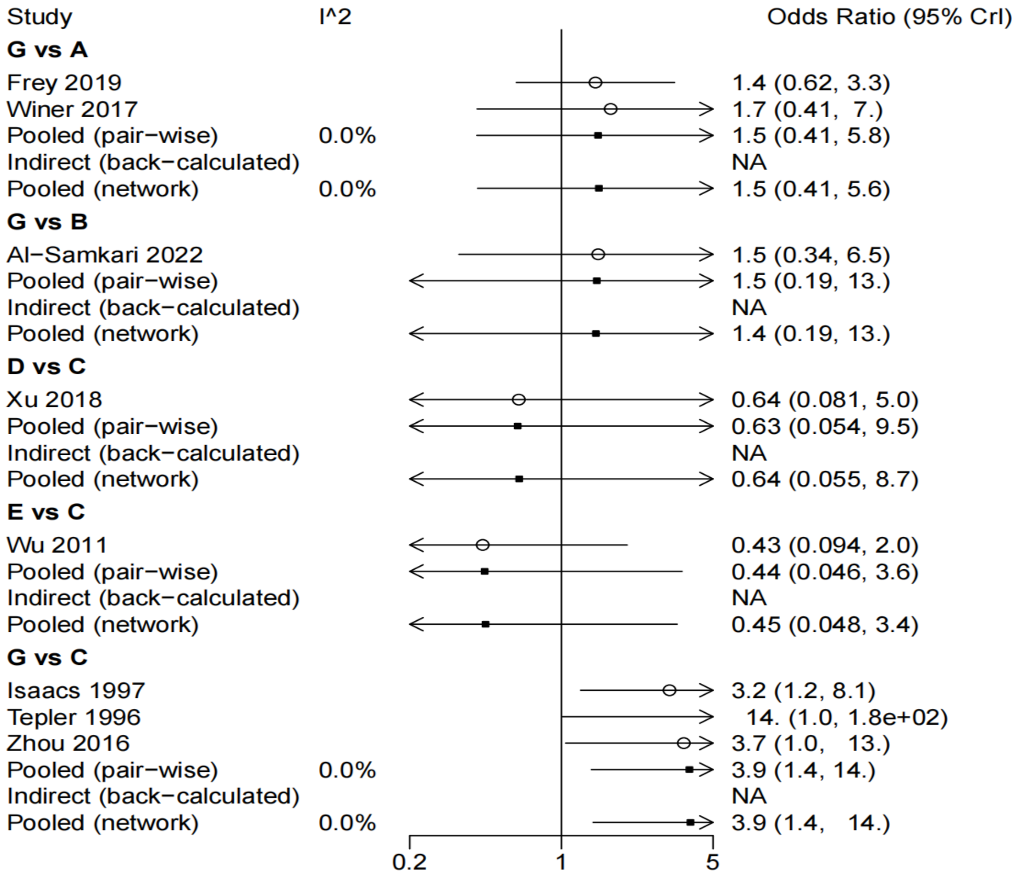

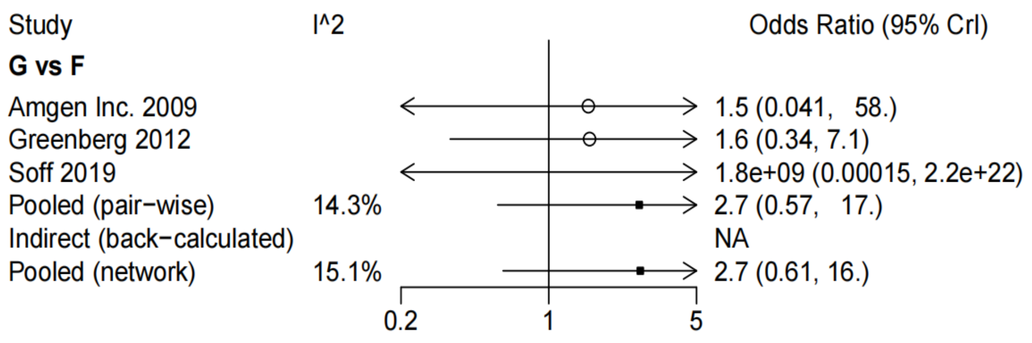

A. Platelet transfusion

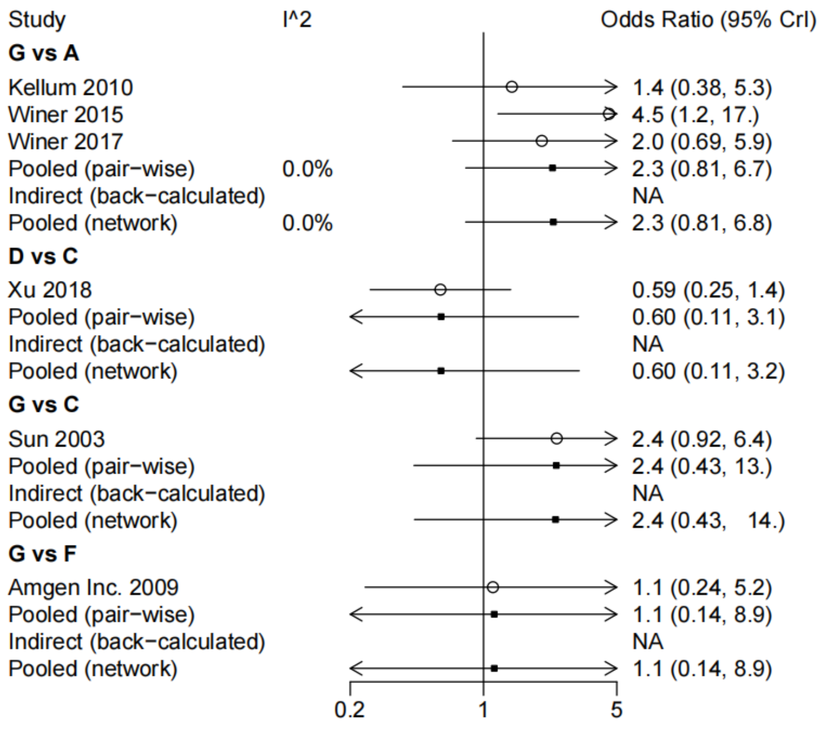

B. Grade 3/4 thrombocytopenia

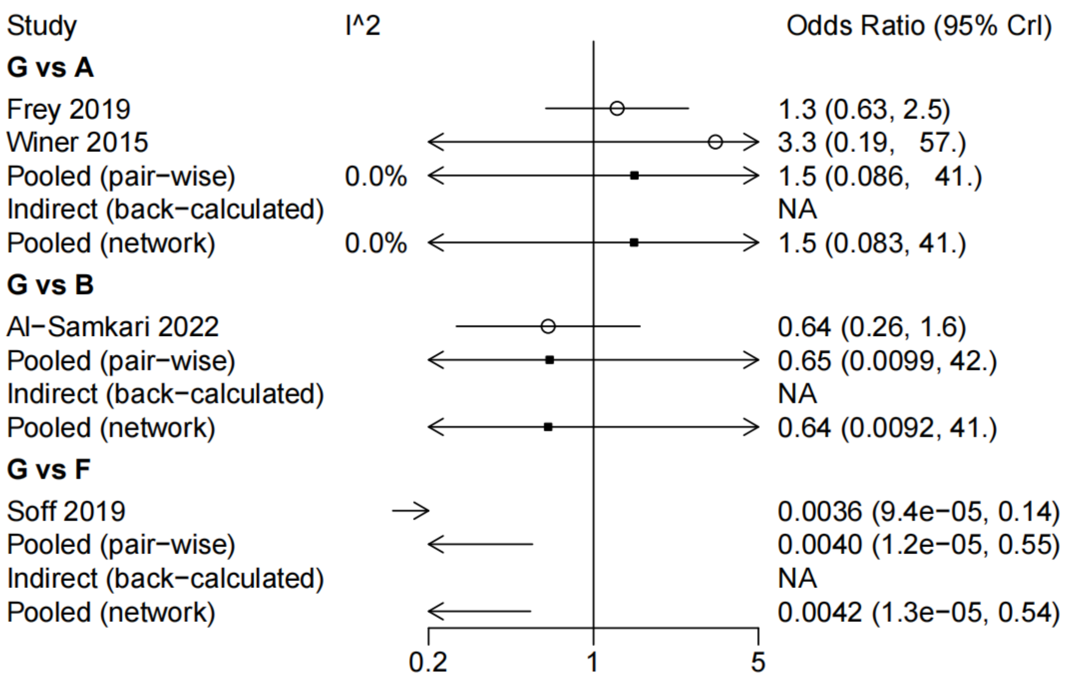

C. Platelet count recovery to ≥100×10^9/L

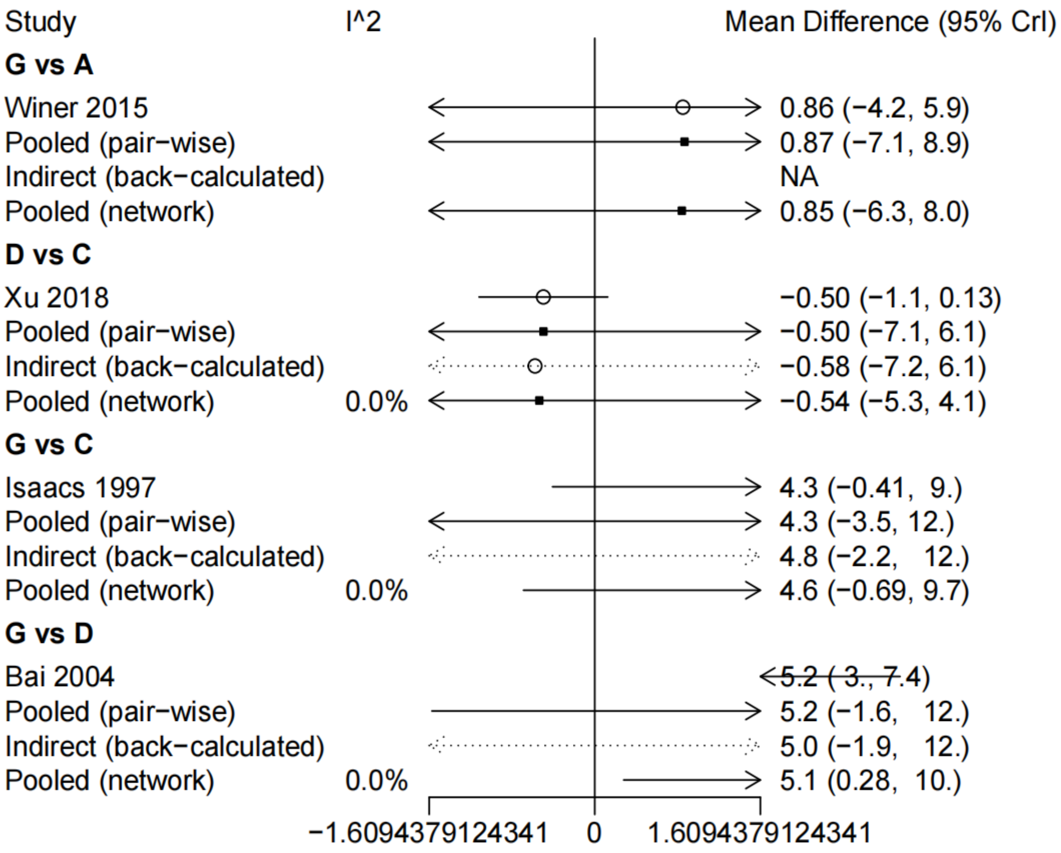

D. Platelet count recovery to ≥100×10^9/L(d)

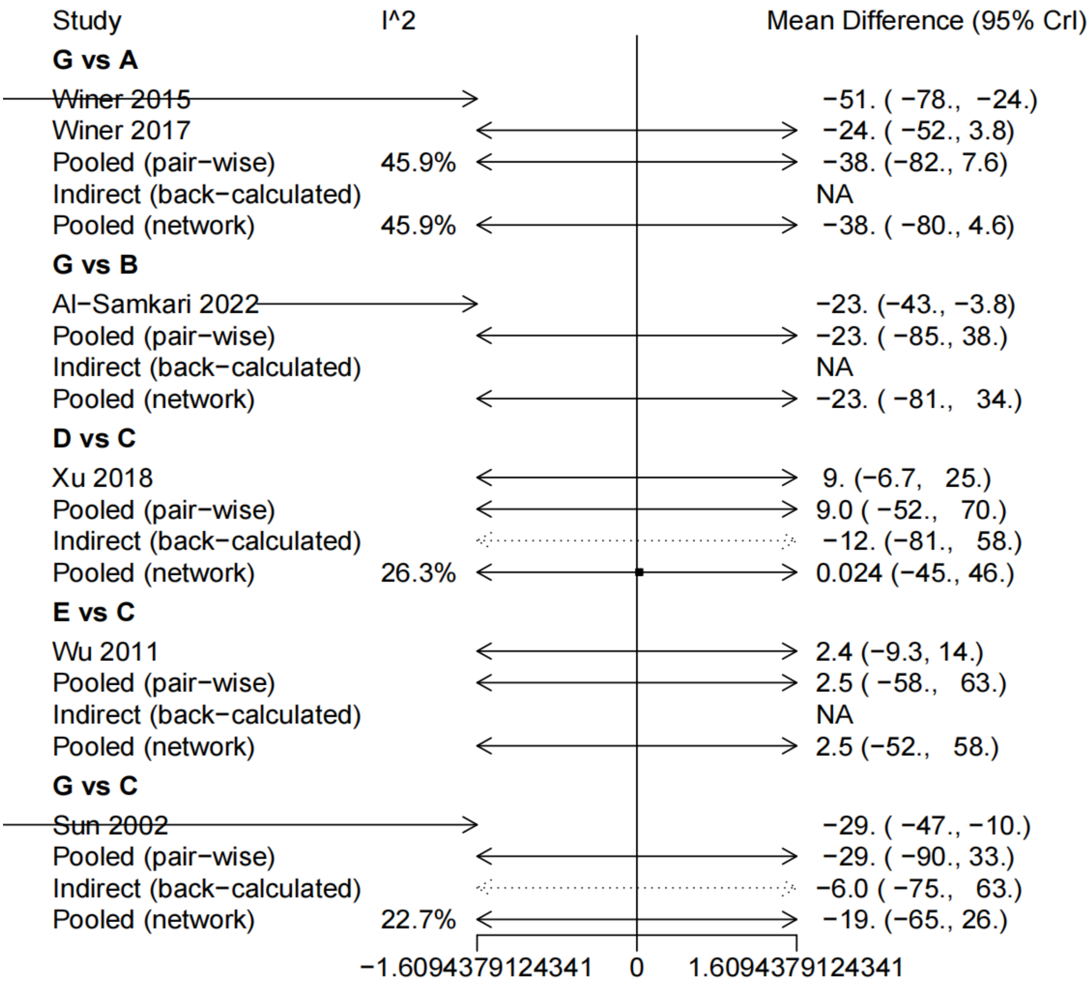

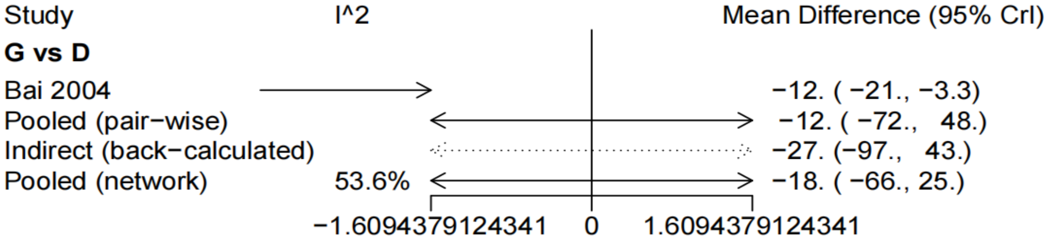

E. Nadir platelet count

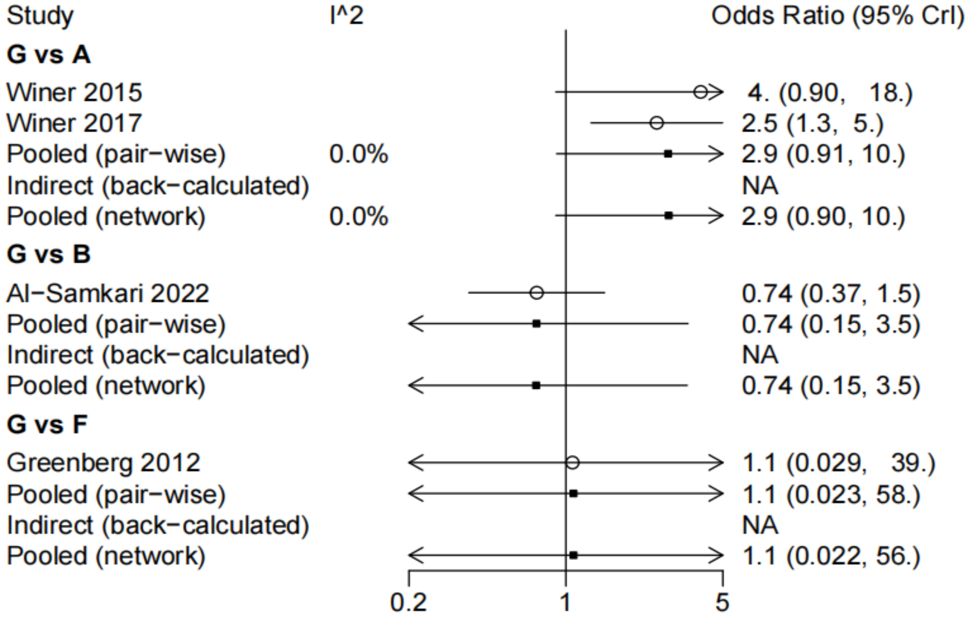

F. Delays/Dose reductions/missed doses due to thrombocytopenia

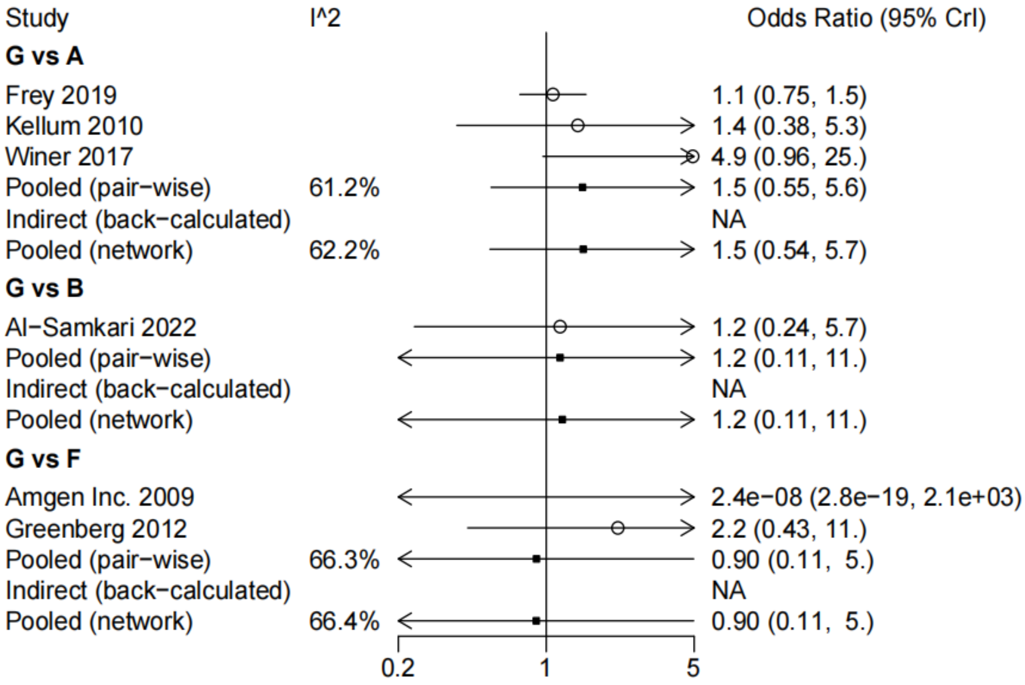

G. Bleeding events

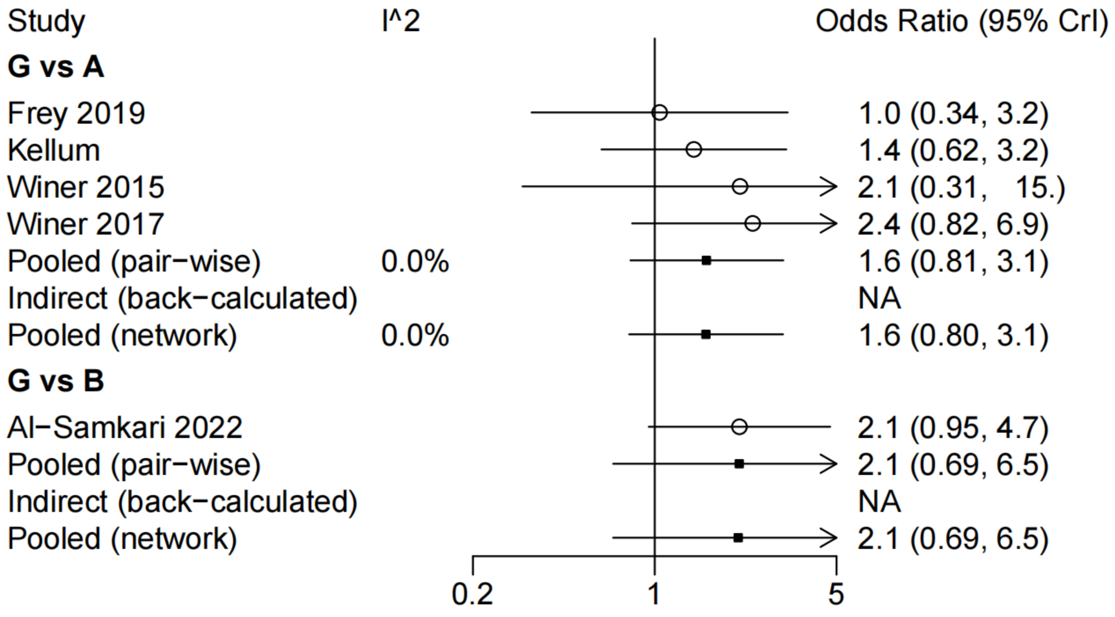

H. Anemia

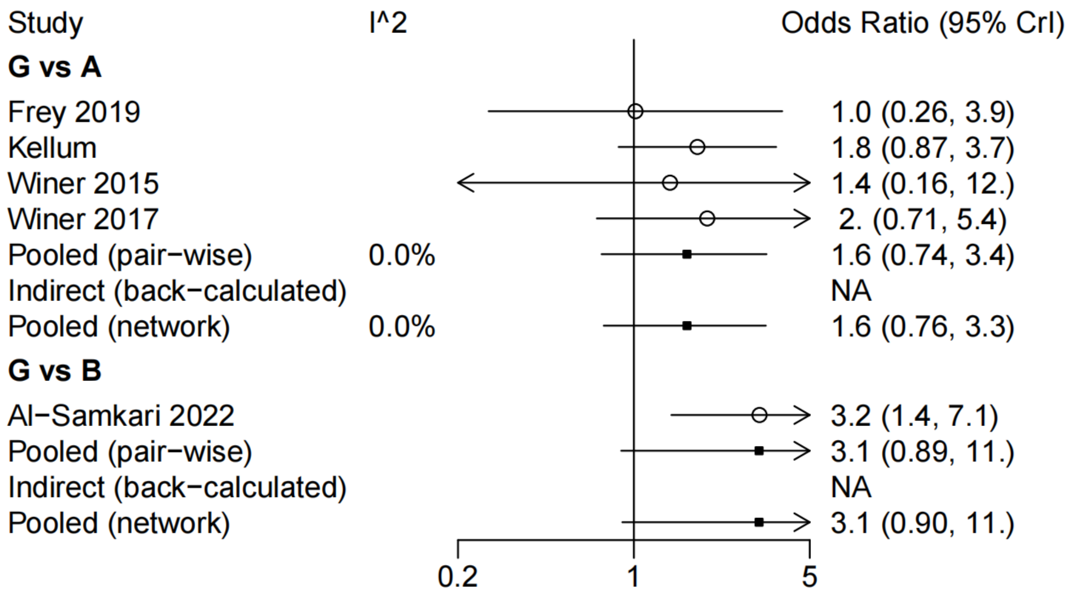

I. Neutropenia

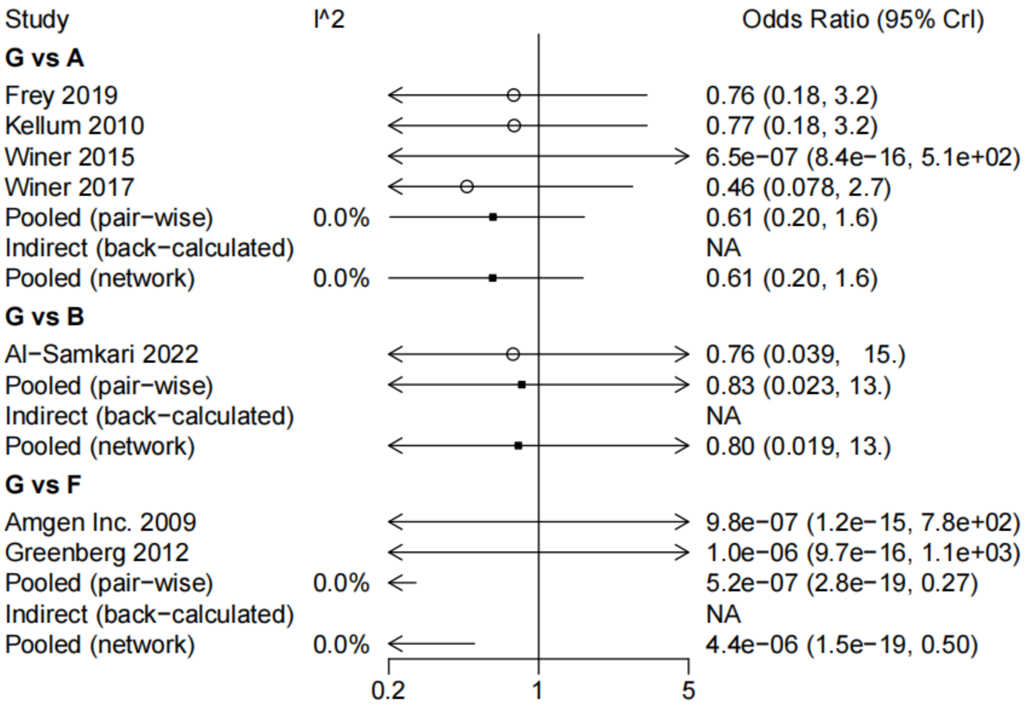

J. Thromboembolic events

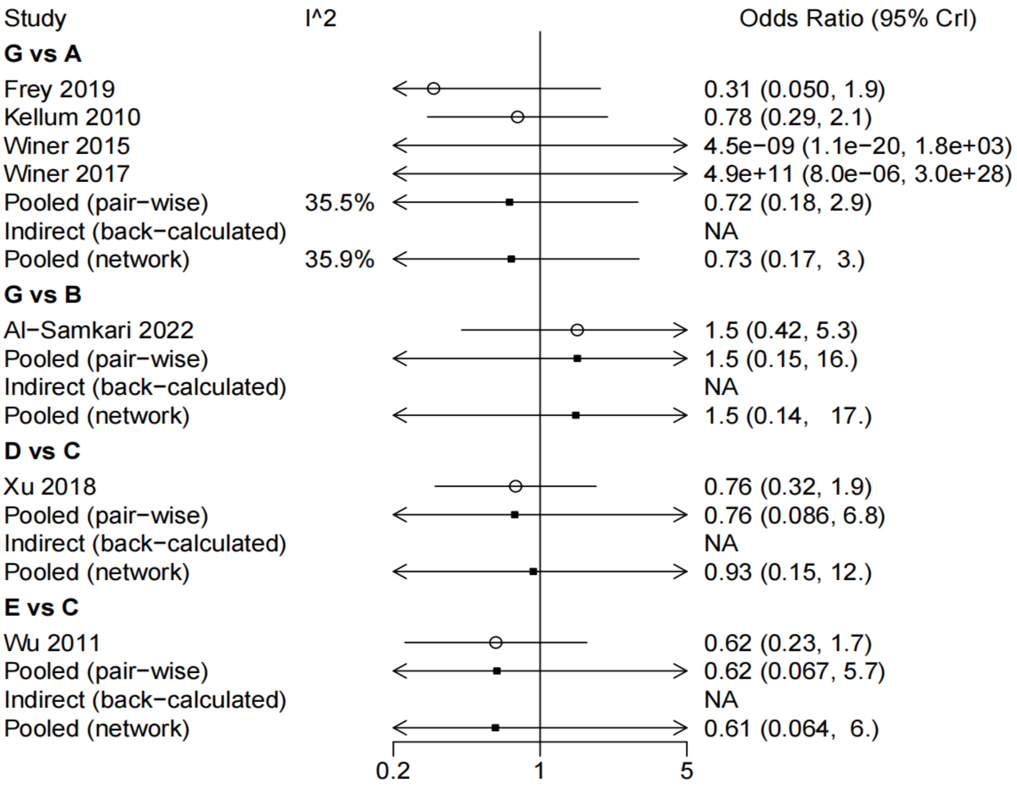

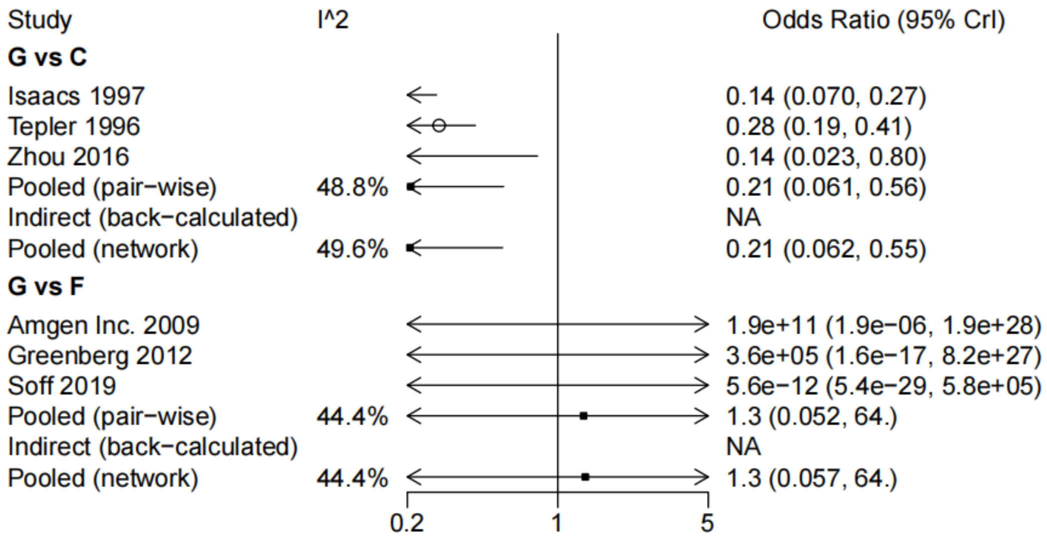

K. Adverse events

Supplementary Figure 3. Forest plots depicting results of head-to-head comparisons according to Bayesian pairwise and network meta-Analyses for platelet transfusion (A), grade 3/4 thrombocytopenia (B), platelet count recovery to ≥100×10^{9} (C), platelet count recovery to ≥100×10^{9}(d) (D), nadir platelet count (E), delays/dose reductions due to thrombocytopenia (F), bleeding events (G), anemia (H), neutropenia (I), thromboembolic events (J), adverse events (K):

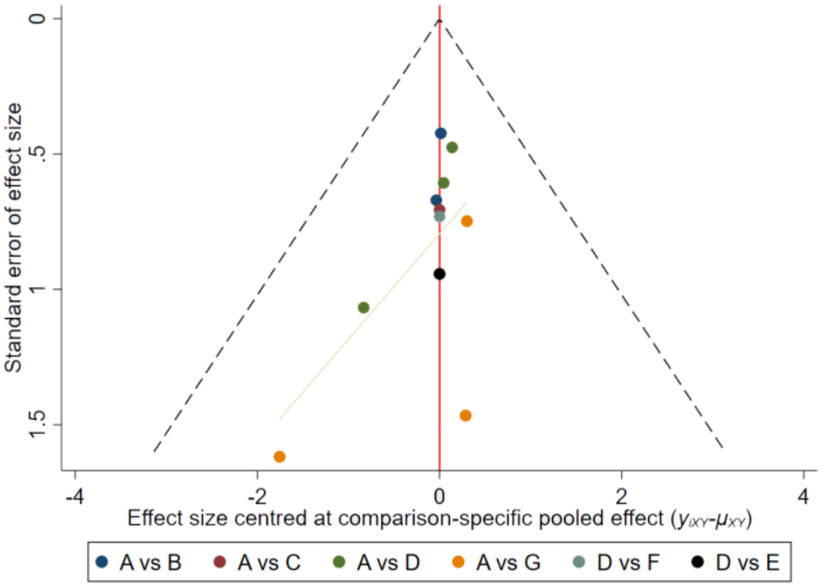

A. **platelet transfusion**

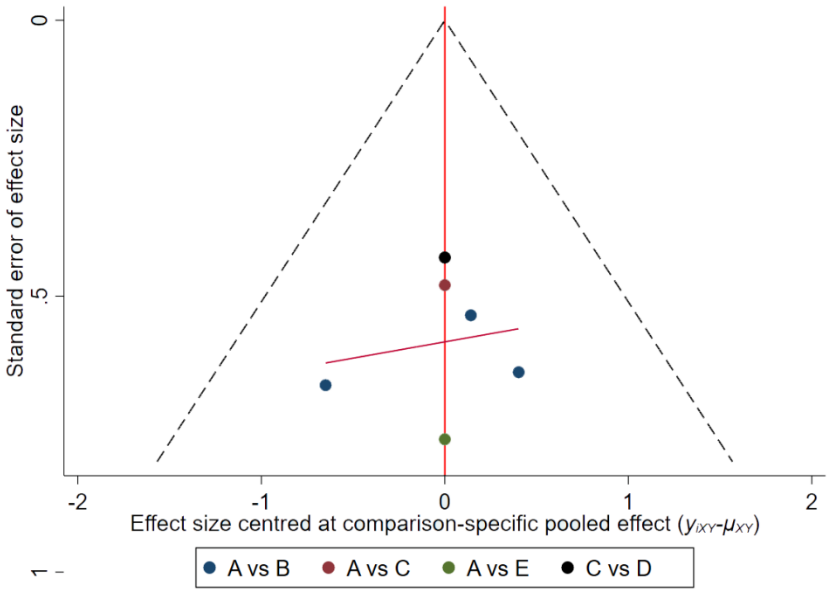

B. Grade 3/4 thrombocytopenia

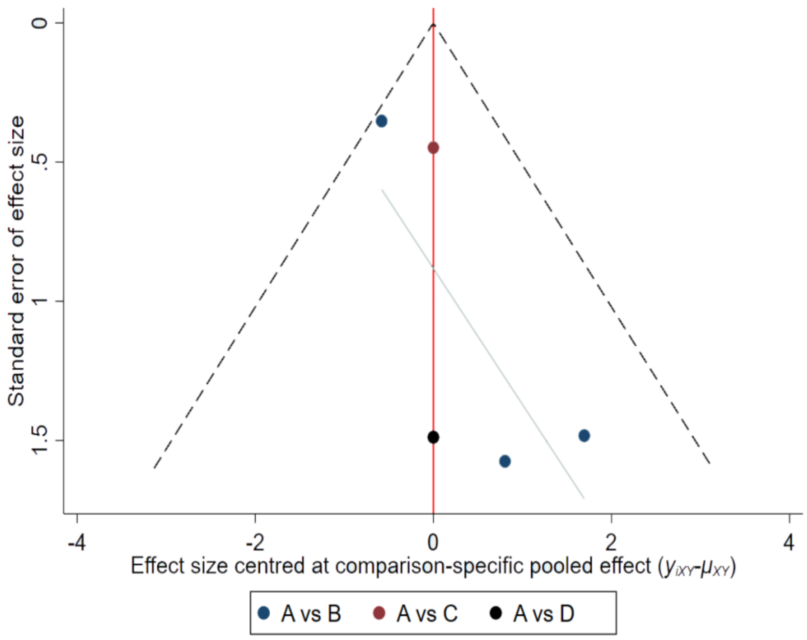

C. Platelet count recovery to >100 x 10^9/L

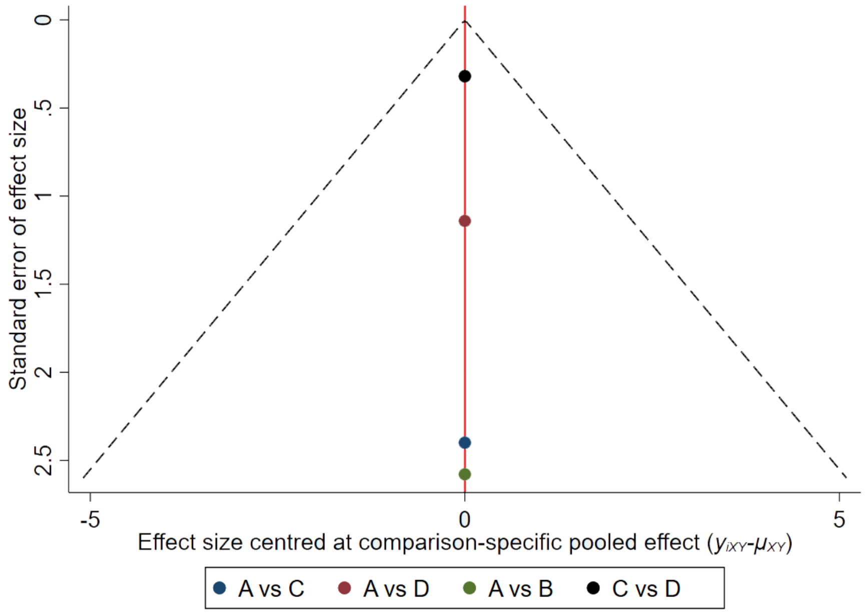

D. Platelet count recovery to 100 x 10^9/L(d)

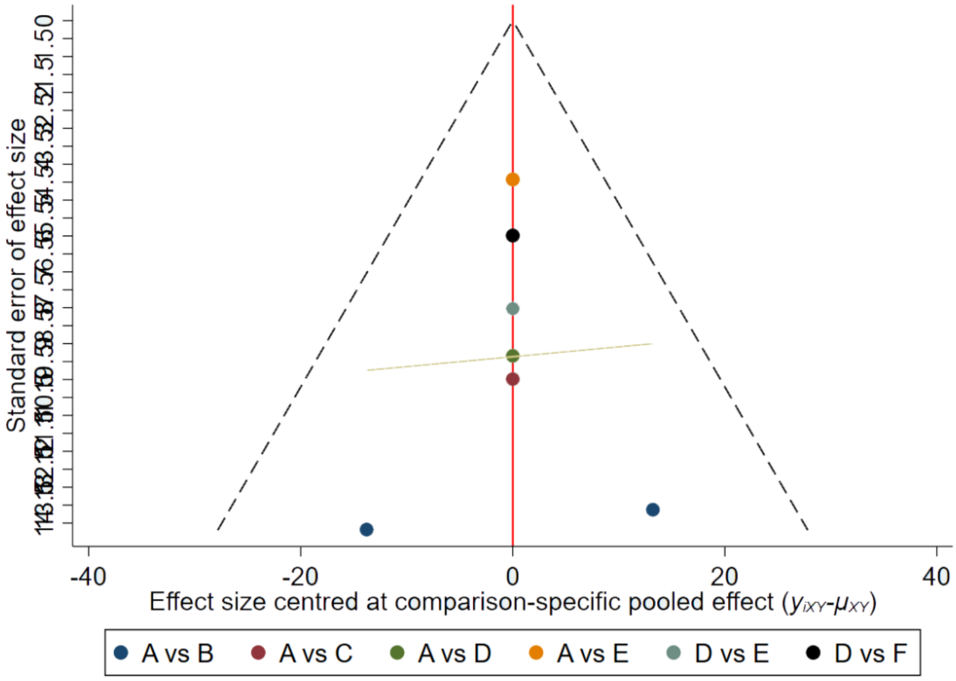

E. Nadir platelet count

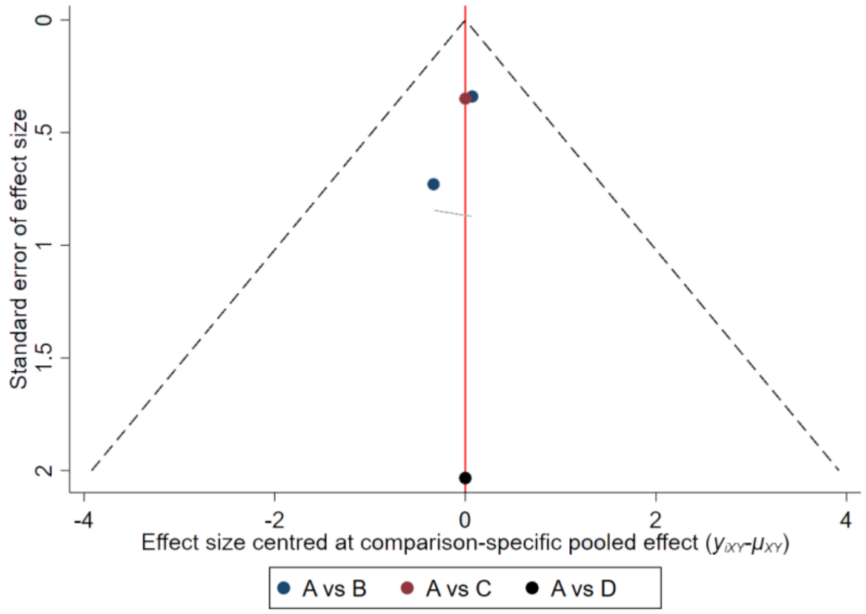

F. Dose delays/dose reductions due to thrombocytopenia

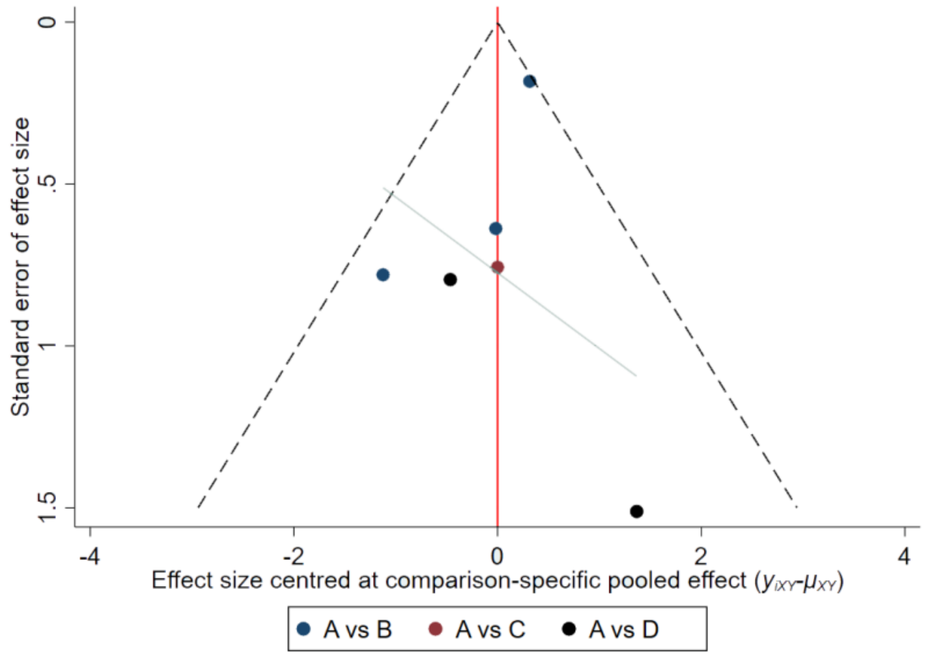

G. Bleeding events

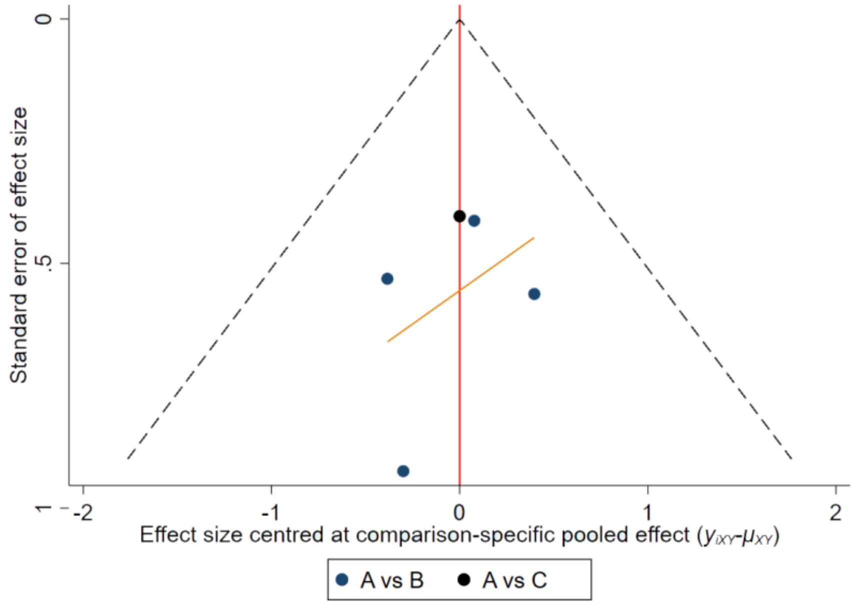

H. Anemia

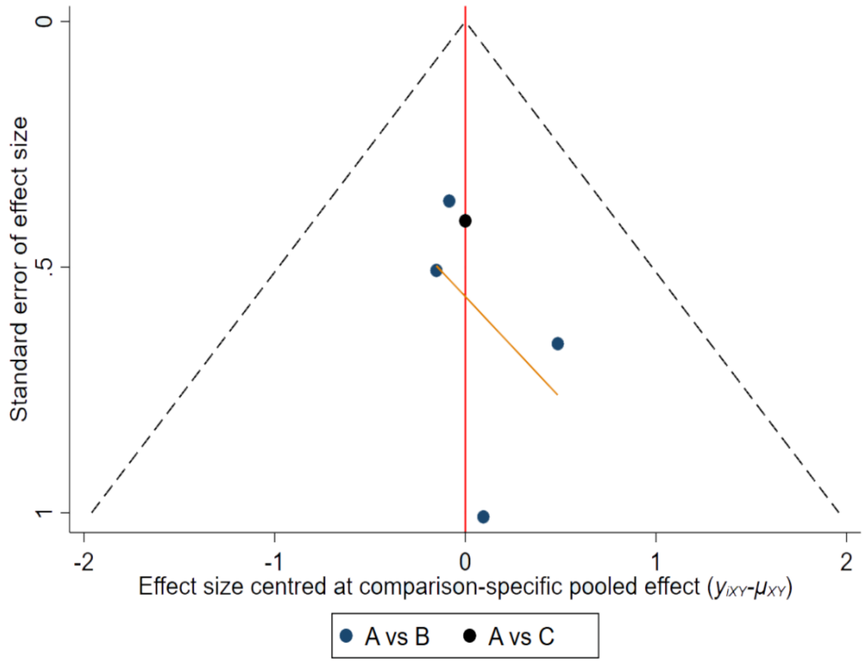

I. Neutropenia

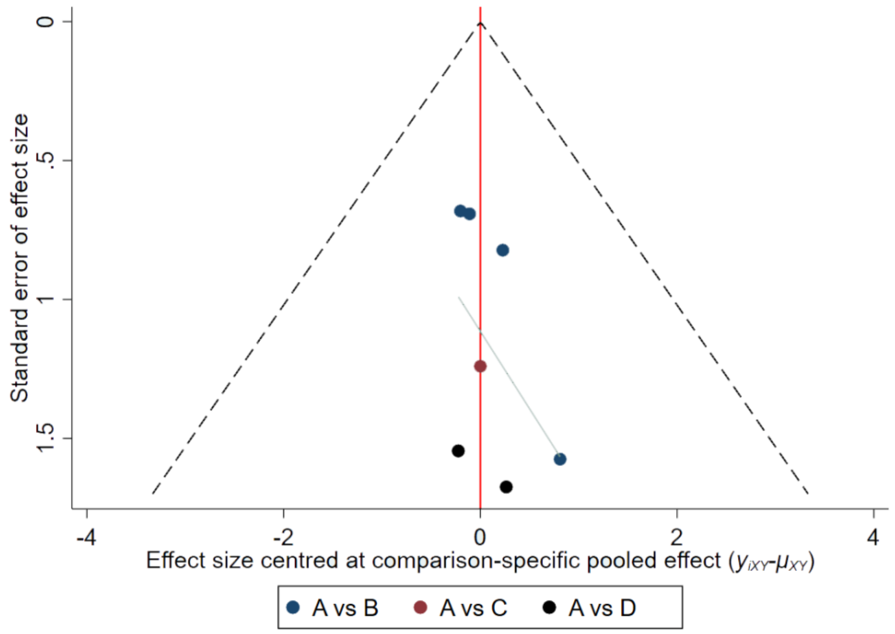

J. Thromboembolic events

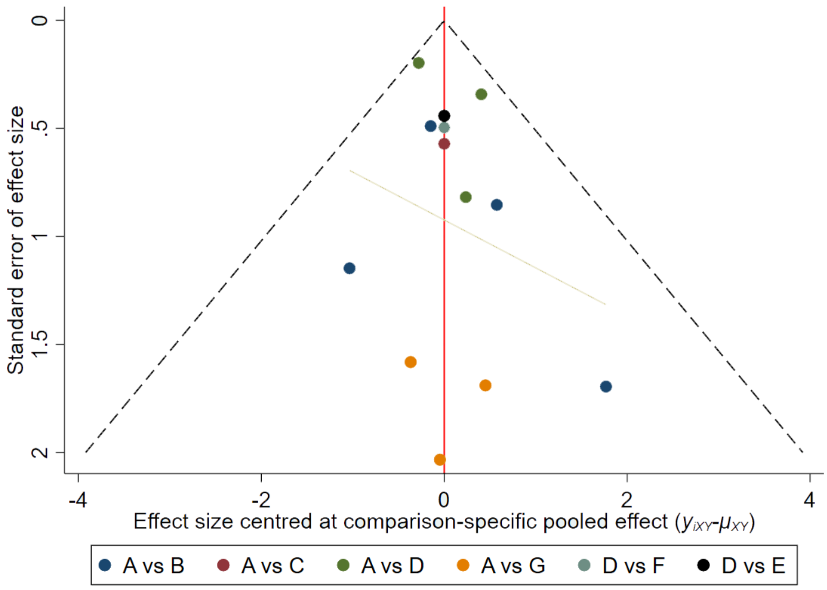

K. Adverse events

Supplementary Figure4. Comparison-adjusted funnel plot. (A)platelet transfusion, (B)grade 3/4 thrombocytopenia, (C)platelet count recovery to $\geq 100\times10^9$, (D)platelet count recovery to $\geq 100\times10^9$(d), (E)nadir platelet count, (F)delays/dose reductions due to thrombocytopenia, (G)bleeding events, (H)anemia, (I)neutropenia, (J)thromboembolic events, (K)adverse events.

A

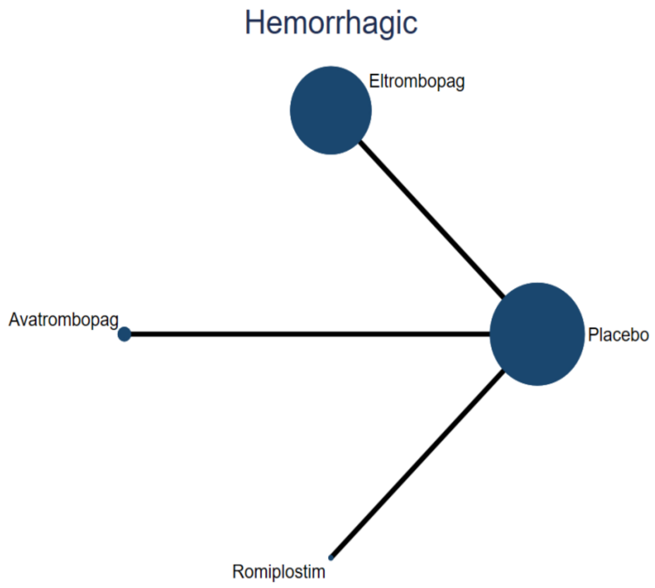

Bleeding events

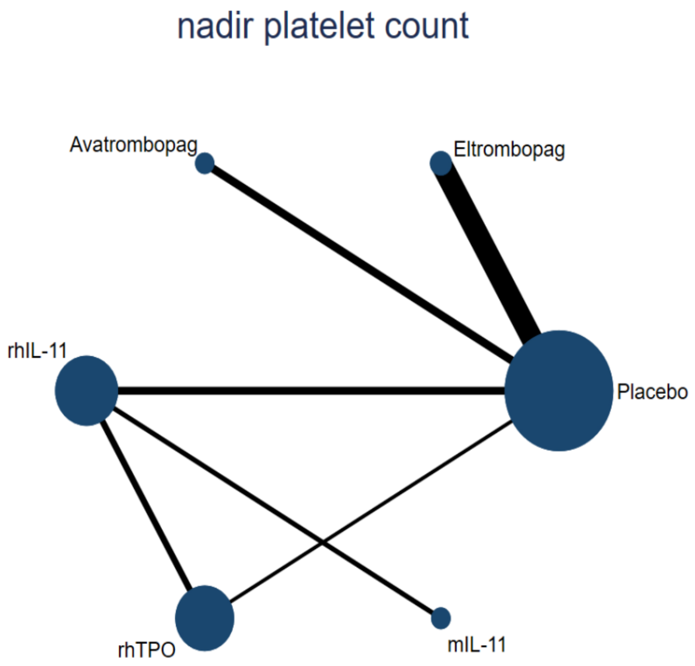

Nadir platelet count

B

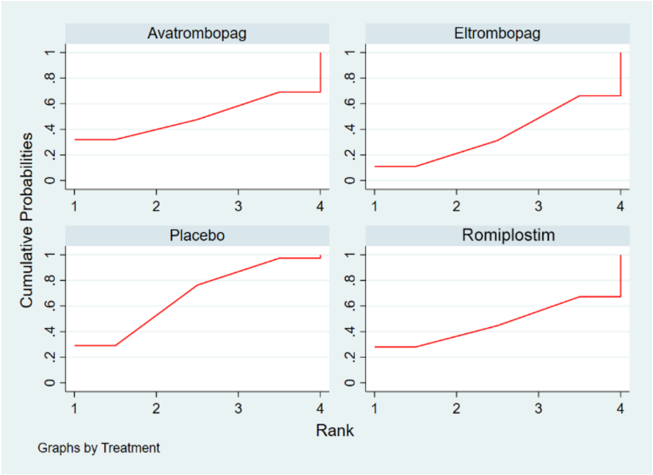

Bleeding events

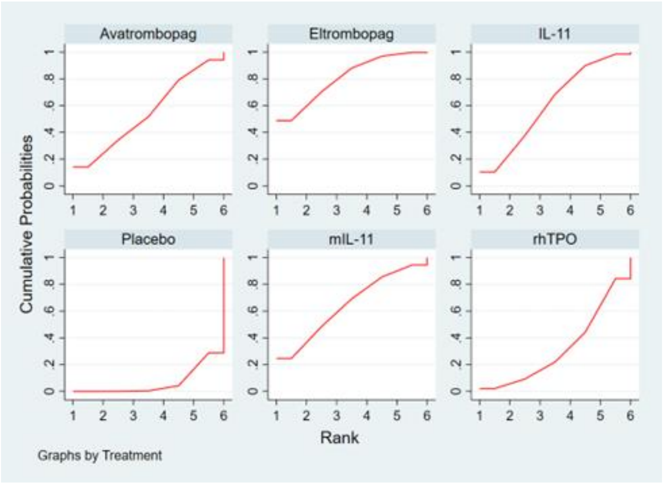

Nadir platelet count

C

Placebo	0.68 (0.27,1.71)	0.80 (0.15,4.39)	0.76 (0.16,3.61)
1.47 (0.59,3.69)	**Eltrombopag**	1.18 (0.17,8.14)	1.12 (0.17,7.39)
1.25 (0.23,6.84)	0.85 (0.12,5.88)	**Avatrombopag**	0.95 (0.10,9.53)
1.31 (0.28,6.19)	0.89 (0.14,5.88)	1.05 (0.10,10.50)	**Romiplostim**

Bleeding events

Placebo	**37.82(12.71,62.93)**	23.40 (-6.61,53.41)	27.49 (-1.40,56.37)	24.30 (-22.49,71.10)	12.00 (-12.35,36.35)
**-37.82(-62.93,-12.71)**	**Eltrombopag**	-14.42(-53.55,24.71)	-10.33(-48.52,27.86)	-25.82 (-60.79,9.16)	-8.11 (-54.04,37.82)
-23.40 (-53.41,6.61)	14.42(-24.71,53.55)	**Avatrombopag**	4.09 (-37.57,45.74)	-11.40 (-50.05,27.25)	6.30 (-42.57,55.17)
-27.49 (-56.37,1.40)	10.33(-27.86,48.52)	-4.09 (-45.74,37.57)	**rhIL-11**	-15.49 (-53.27,22.29)	2.22 (-23.37,27.81)
-24.30 (-71.10,22.49)	25.82(-9.16,60.79)	11.40 (-27.25,50.05)	15.49 (-22.29,53.27)	**rhTPO**	17.70 (-27.91,63.31)
-12.00 (-36.35,12.35)	8.11 (-37.82,54.04)	-6.30 (-55.17,42.57)	-2.22 (-27.81,23.37)	-17.70 (-63.31,27.91)	**mIL-11**

Nadir platelet count

D

Treatment	SUCRA	MeanRank
Placebo	67.6	2.0
Eltrombopag	35.7	2.9
Avatrombopag	49.7	2.5
Romiplostim	47.0	2.6

Bleeding events

Treatment	SUCRA	MeanRank
Placebo	6.7	5.7
Eltrombopag	80.8	2.0
Avatrombopag	54.7	3.3
rhIL-11	61.0	2.9
rhTPO	32.4	4.4
mIL-11	64.5	2.8

Nadir platelet count

Supplementary Figure 5. Sensitive analysis of network meta-analysis on bleeding events and nadir platelet count. (A) Network diagram for the sensitivity analysis on bleeding events and nadir platelet count. (B) Sucra for the sensitivity analysis on bleeding events and nadir platelet count. (C) Pooled ORs (95% credible intervals) for the sensitivity analysis on bleeding events and nadir platelet count. (D) Bayesian ranking results of the sensitivity analysis on bleeding events and nadir platelet count.

A

**Placebo**	1.57 (0.67,3.70)	1.20 (0.34,4.27)	**4.59 (2.56,8.22)**	**3.49 (1.05,11.64)**	2.87 (0.80,10.36)	1.12 (0.15,8.27)
1.45 (0.72,2.92)	**Eltrombopag**	0.76 (0.17,3.54)	**2.92 (1.05,8.15)**	2.23 (0.51,9.68)	1.83 (0.39,8.49)	0.71 (0.08,6.29)
0.75 (0.19,2.99)	0.52 (0.11,2.45)	**Avatrombopag**	**3.82 (1.00,15.46)**	2.91 (0.51,16.75)	2.39 (0.39,14.55)	0.93 (0.09,9.97)
**3.58 (1.53,8.38)**	2.47 (0.82,7.46)	4.77 (0.94,24.23)	**rhIL-11**	0.76 (0.27,2.18)	0.63 (0.20,1.96)	0.24 (0.03,1.96)
6.11 (0.80,46.70)	4.22 (0.49,36.34)	8.14 (0.69,95.37)	1.71 (0.27,10.83)	**rhTPO**	0.82 (0.17,3.89)	0.32 (0.03,3.31)
**7.54 (1.42,39.93)**	5.21 (0.85,31.82)	**10.05 (1.15,87.76)**	2.11 (0.50,8.83)	1.23 (0.12,12.80)	**mIL-11**	0.39 (0.04,4.19)
2.06 (0.62,6.89)	1.42 (0.35,5.75)	2.74 (0.44,17.23)	0.58 (0.13,2.52)	0.34 (0.03,3.59)	0.27 (0.03,2.14)	**Romiplostim**

B

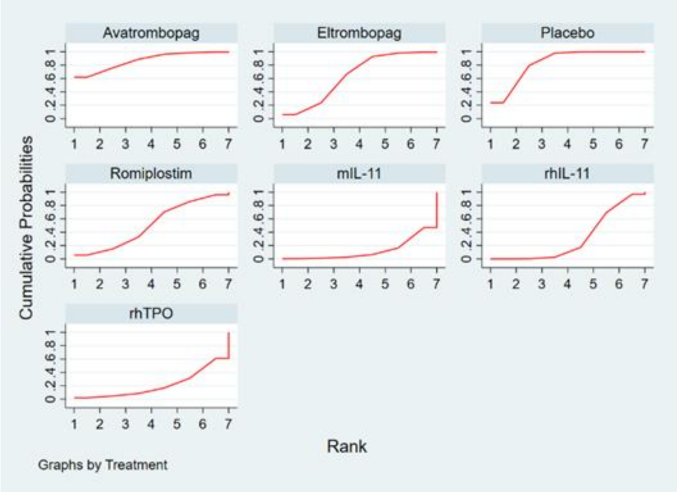

Platelet transfusion

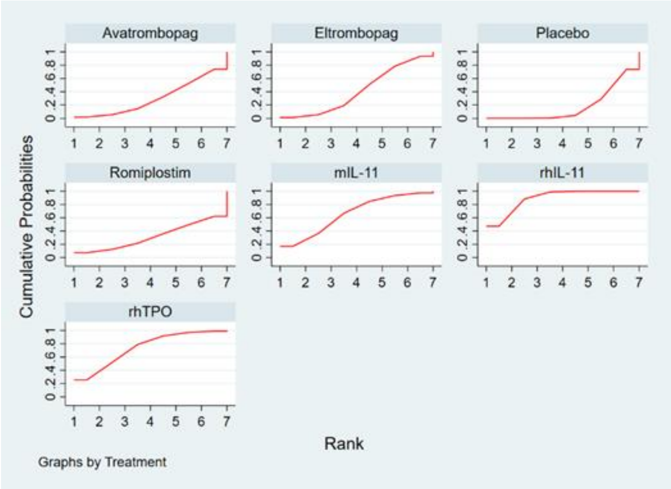

Adverse events

C

Treatment	SUCRA	PrBest	MeanRank
Placebo	83.5	23.8	2.0
Eltrombopag	64.4	6.0	3.1
Avatrombopag	86.9	62.0	1.8
rhIL-11	31.0	0.0	5.1
rhTPO	20.8	2.1	5.8
mIL-11	12.3	0.4	6.3
Romiplostim	51.0	5.7	3.9

Platelet transfusion

Treatment	SUCRA	PrBest	MeanRank
Placebo	17.9	0.0	5.9
Eltrombopag	41.5	1.2	4.5
Avatrombopag	30.1	1.9	5.2
rhIL-11	89.1	47.2	1.7
rhTPO	74.1	25.4	2.6
mIL-11	66.0	16.9	3.0
Romiplostim	31.5	7.3	5.1

Adverse events

Supplementary Figure 6. Sensitive analysis of network meta-analysis with the exclusion of a pediatric study. (A) Pooled ORs (95% credible intervals) for adverse events in the upper triangle and platelet transfusion in the lower triangle. (B) Sucra for the sensitivity analysis on platelet transfusion and adverse events. (C) Bayesian ranking results of the sensitivity analysis on platelet transfusion and adverse events.

A

**Placebo**	1.33 (0.49,3.61)	1.20 (0.33,4.40)	**4.63 (2.50,8.56)**	**3.52 (1.01,12.31)**	2.90 (0.77,10.92)	1.14 (0.11,11.47)
1.45 (0.72,2.92)	**Eltrombopag**	0.90 (0.18,4.64)	**3.47 (1.08,11.17)**	2.64 (0.54,13.06)	2.18 (0.41,11.41)	0.86 (0.07,10.59)
0.75 (0.19,2.99)	0.50 (0.07,3.38)	**Avatrombopag**	**3.85 (1.00,16.24)**	2.93 (0.48,17.83)	2.41 (0.38,15.47)	0.95 (0.07,13.44)
**3.50 (1.75,6.98)**	2.39 (0.50,11.43)	4.77 (0.94,24.23)	**rhIL-11**	0.76 (0.26,2.26)	0.63 (0.19,2.03)	0.25 (0.02,2.69)
5.97 (0.83,42.92)	4.08 (0.36,45.93)	8.14 (0.69,95.37)	1.71 (0.27,10.83)	**rhTPO**	0.82 (0.17,4.08)	0.32 (0.02,4.47)
**7.37 (1.50,36.18)**	5.03 (0.60,42.03)	**10.05 (1.15,87.76)**	2.11 (0.50,8.83)	1.23 (0.12,12.80)	**mIL-11**	0.39 (0.03,5.64)
2.06 (0.62,6.89)	2.59 (0.21,31.64)	5.18 (0.41,65.64)	1.09 (0.11,10.75)	0.64 (0.03,12.09)	0.52 (0.03,7.70)	**Romiplostim**

B

**Placebo**	0.42 (0.14,1.27)	0.80 (0.16,4.09)	0.75 (0.16,3.59)
0.67 (0.25,1.77)	**Eltrombopag**	1.91 (0.27,13.74)	1.79 (0.25,12.92)
1.03 (0.09,11.66)	1.54 (0.11,21.09)	**Avatrombopag**	0.94 (0.10,8.98)
0.54 (0.03,11.21)	0.81 (0.03,19.58)	0.53 (0.01,25.71)	**Romiplostim**

C

**Placebo**	**0.52 (0.30,0.91)**	**0.32 (0.15,0.72)**
**1.75 (1.01,3.20)**	**Eltrombopag**	0.62 (0.23,1.63)
**2.08 (1.00,4.58)**	1.19 (0.44,3.20)	**Avatrombopag**

D

**Placebo**	**0.37 (0.20,0.68)**	1.33 (0.67,2.63)	
0.20 (0.02,1.63)	**Eltrombopag**	**3.56 (1.43,8.88)**	
0.66 (0.27,1.59)	3.38 (0.34,33.36)	**Avatrombopag**	
**0.01 (0.00,0.19)**	0.05 (0.00,1.91)	**0.02 (0.00,0.32)**	**Romiplostim**

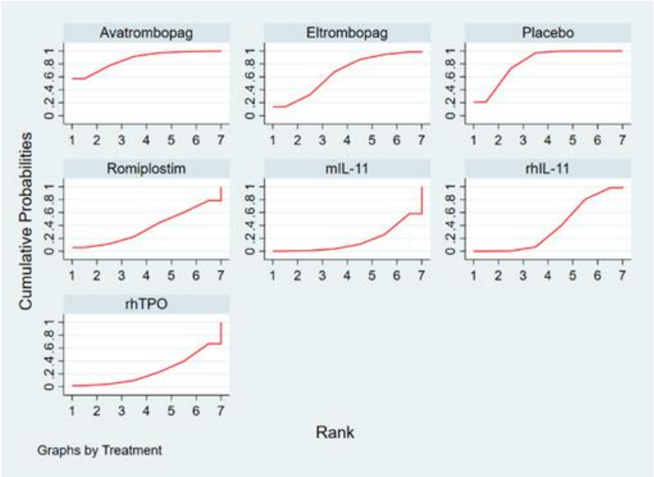

E. Platelet transfusion

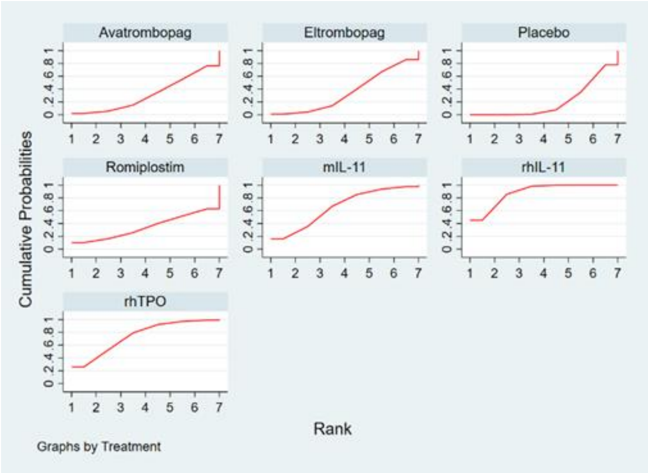

F. Adverse events

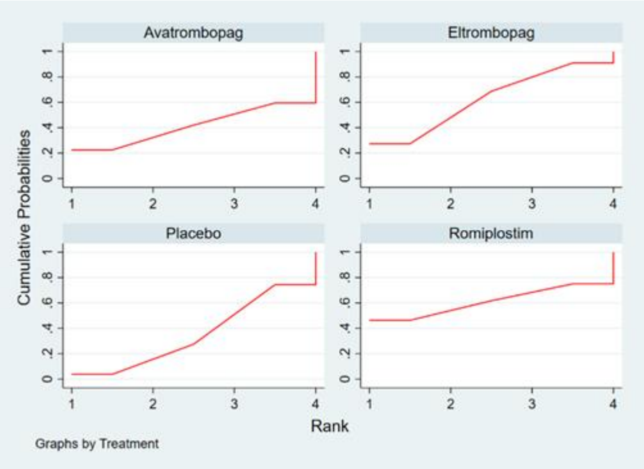

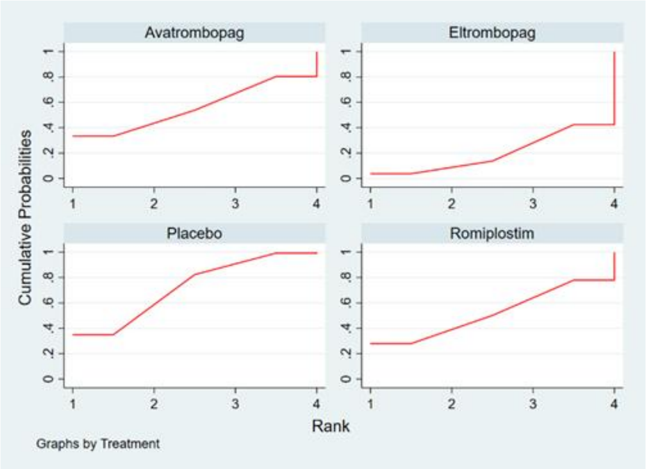

G. Thrombosis

H. Bleeding events

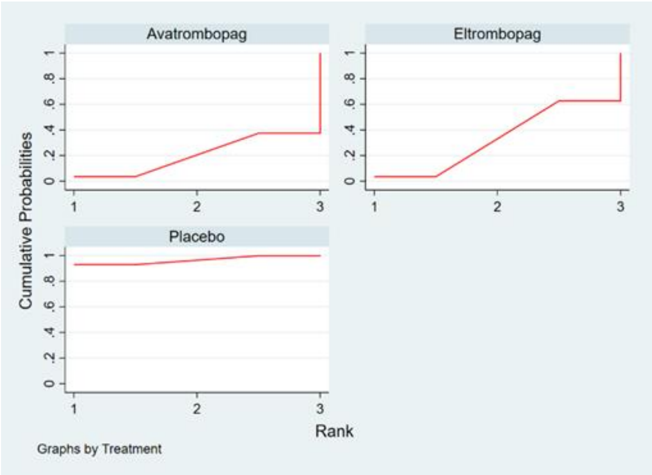

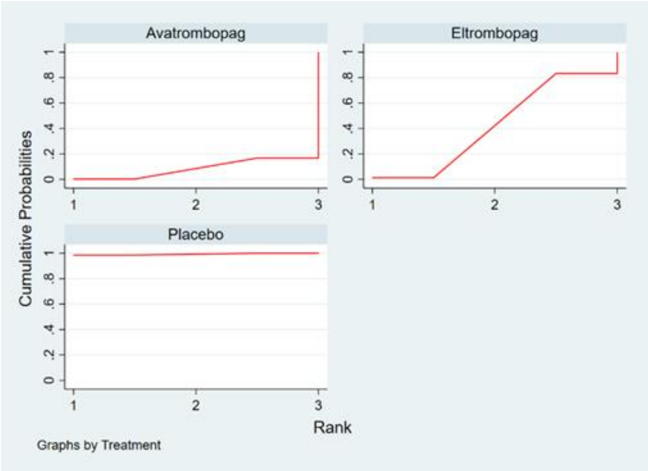

I. Anemia

J. Neutropenia

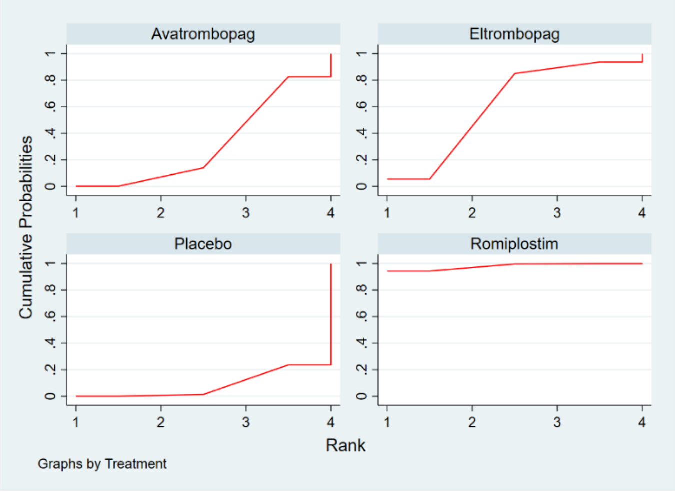

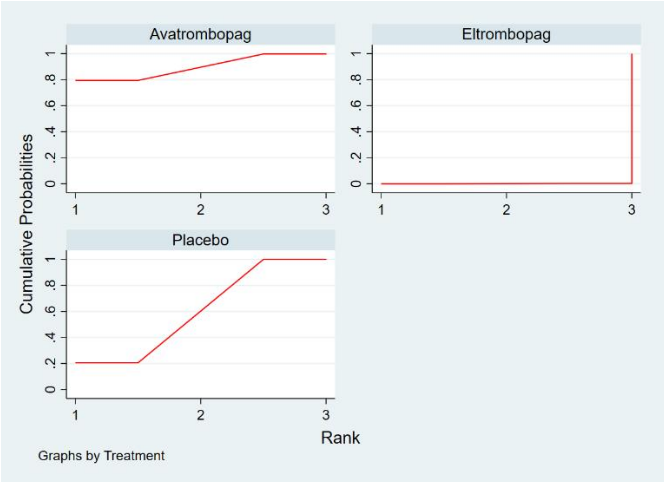

K. PLT recovery to ≥100×10^9/L

L. Dose delays/dose reductions

Treatment	SUCRA	PrBest	MeanRank
Placebo	81.9	21.0	2.1
Eltrombopag	65.8	13.8	3.1
Avatrombopag	87.1	57.2	1.8
rhIL-11	37.4	0.0	4.8
rhTPO	24.2	1.9	5.5
mIL-11	16.6	0.3	6.0
Romiplostim	37.0	5.8	4.8

Treatment	SUCRA	PrBest	MeanRank
Placebo	20.2	0.0	5.8
Eltrombopag	35.4	0.9	4.9
Avatrombopag	31.5	2.0	5.1
rhIL-11	88.2	45.2	1.7
rhTPO	74.4	25.9	2.5
mIL-11	65.8	15.9	3.0
Romiplostim	34.5	10.1	4.9

M. Platelet transfusion

Treatment	SUCRA	PrBest	MeanRank
Placebo	72.2	35.0	1.8
Eltrombopag	20.0	3.8	3.4
Avatrombopag	55.8	33.3	2.3
Romiplostim	52.0	27.9	2.4

N. Adverse events

Treatment	SUCRA	PrBest	MeanRank
Placebo	35.2	3.8	2.9
Eltrombopag	62.4	27.4	2.1
Avatrombopag	41.3	22.5	2.8
Romiplostim	61.0	46.3	2.2

O. Bleeding events

Treatment	SUCRA	PrBest	MeanRank
Placebo	99.2	98.5	1.0
Eltrombopag	42.3	1.2	2.2
Avatrombopag	8.5	0.3	2.8

P. Thrombosis

Treatment	SUCRA	PrBest	MeanRank
Placebo	96.6	93.4	1.1
Eltrombopag	33.1	3.5	2.3
Avatrombopag	20.3	3.2	2.6

Q. Neutropenia

Treatment	SUCRA	PrBest	MeanRank
Placebo	8.3	0.0	3.8
Eltrombopag	61.5	5.6	2.2
Avatrombopag	32.3	0.1	3.0
Romiplostim	97.9	94.3	1.1

R. Anemia

Treatment	SUCRA	PrBest	MeanRank
Placebo	60.3	20.6	1.8
Eltrombopag	0.2	0.0	3.0
Avatrombopag	89.6	79.4	1.2

S. PLT recovery to ≥100×10^9/L

T. Dose delays/dose reductions

Supplementary Figure 7. Sensitive analysis of network meta-analysis after excluding the three studies on hematologic malignancies. (A) Pooled ORs (95% credible intervals) for adverse events in the upper triangle and platelet transfusion in the lower triangle. (B) Pooled ORs (95% credible intervals) for thrombosis in the upper triangle and bleeding event in the lower triangle. (C) Pooled ORs (95% credible intervals) for neutropenia in the upper triangle and anemia in the lower triangle. (D) Pooled ORs (95% credible intervals) for dose delays/dose reductions in the upper triangle and PLT recovery to ≥100×10^9/L in the lower triangle. (E) Sucra for the sensitivity analysis on platelet transfusion. (F) Sucra for the sensitivity analysis on adverse events. (G) Sucra for the sensitivity analysis on thrombosis. (H) Sucra for the sensitivity analysis on bleeding events. (I) Sucra for the sensitivity analysis on neutropenia. (J) Sucra for the sensitivity analysis on anemia. (K) Sucra for the sensitivity analysis on PLT recovery to ≥100×10^9/L. (L) Sucra for the sensitivity analysis on dose delays/dose reductions. (M) Bayesian ranking results of the sensitivity analysis on platelet transfusion. (N) Bayesian ranking results of the sensitivity analysis on adverse events. (O) Bayesian ranking results of the sensitivity analysis on thrombosis. (P) Bayesian ranking results of the sensitivity analysis on bleeding events. (Q) Bayesian ranking results of the sensitivity analysis on neutropenia. (R) Bayesian ranking results of the sensitivity analysis on anemia. (S) Bayesian ranking results of the sensitivity analysis on PLT recovery to ≥100×10^9/L. (T) Bayesian ranking results of the sensitivity analysis on dose delays/dose reductions.

A.

**Placebo**	1.97 (0.53,7.29)	**4.73 (2.65,8.44)**	**3.60 (1.06,12.20)**	2.96 (0.81,10.84)	0.78 (0.03,18.36)
1.45 (0.72,2.92)	**Eltrombopag**	2.40 (0.57,10.07)	1.83 (0.31,10.96)	1.50 (0.24,9.52)	0.39 (0.01,12.11)
**3.50 (1.75,6.98)**	2.42 (0.90,6.48)	**rhIL-11**	0.76 (0.26,2.23)	0.63 (0.20,2.00)	0.16 (0.01,4.10)
5.97 (0.83,42.92)	4.13 (0.51,33.51)	1.71 (0.27,10.83)	**rhTPO**	0.82 (0.17,4.00)	0.22 (0.01,6.41)
**7.37 (1.50,36.18)**	5.09 (0.89,29.01)	2.11 (0.50,8.83)	1.23 (0.12,12.80)	**mIL-11**	0.26 (0.01,8.01)
1.55 (0.09,27.36)	1.07 (0.06,20.59)	0.44 (0.02,8.50)	0.26 (0.01,8.46)	0.21 (0.01,5.60)	**Romiplostim**

B.

**Placebo**		
0.63 (0.24,1.69)	**Eltrombopag**	
0.54 (0.03,11.21)	0.86 (0.04,20.69)	**Romiplostim**

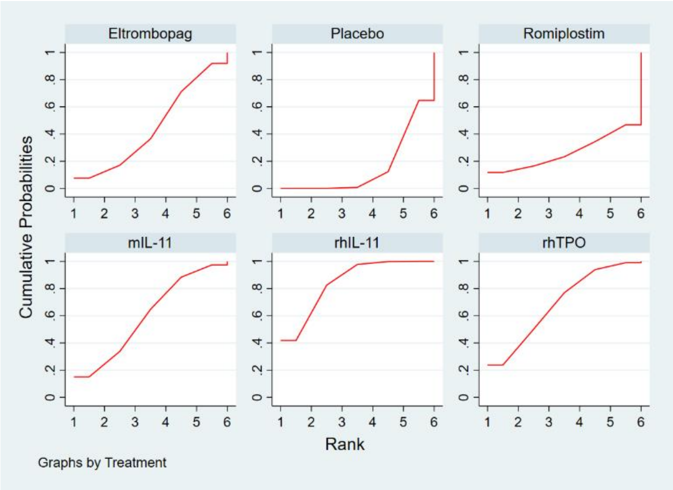

C. Adverse events

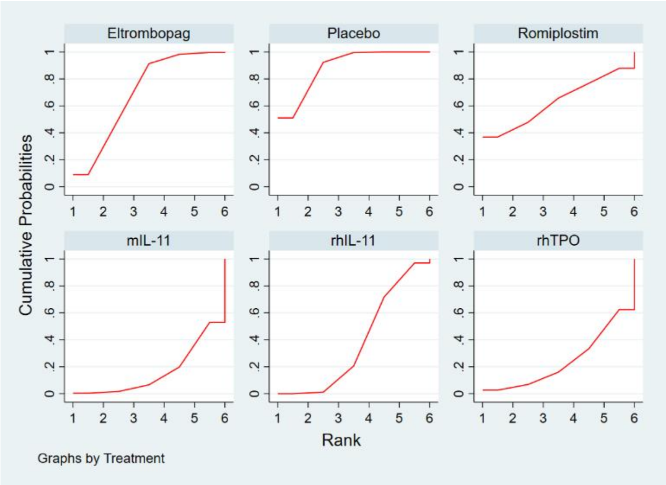

D. Platelet transfusion

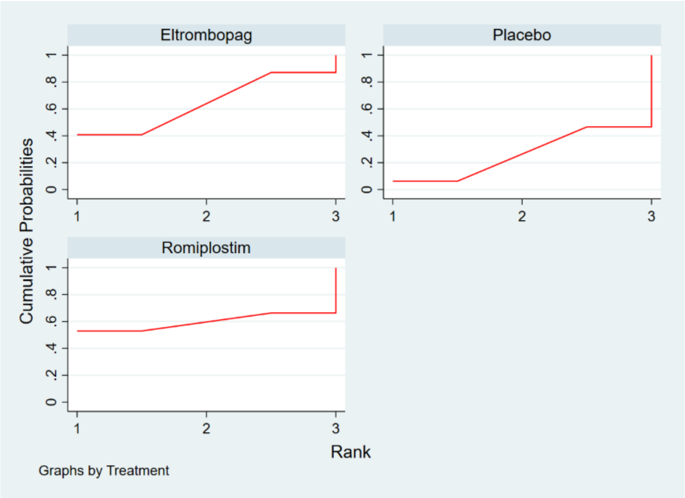

E. Thrombosis

Treatment	SUCRA	PrBest	MeanRank
Placebo	15.6	0.0	5.2
Eltrombopag	44.8	7.6	3.8
rhIL-11	84.4	41.8	1.8
rhTPO	68.8	23.8	2.6
mIL-11	59.9	15.1	3.0
Romiplostim	26.6	11.8	4.7

Treatment	SUCRA	PrBest	MeanRank
Placebo	88.6	51.1	1.6
Eltrombopag	69.7	9.0	2.5
rhIL-11	38.1	0.0	4.1
rhTPO	24.3	2.7	4.8
mIL-11	16.3	0.4	5.2
Romiplostim	63.0	36.9	2.8

F. Adverse events

Treatment	SUCRA	PrBest	MeanRank
Placebo	26.4	6.3	2.5
Eltrombopag	63.9	40.8	1.7
Romiplostim	59.6	52.9	1.8

G. Platelet transfusion

H. Thrombosis

Supplementary Figure 8. Sensitive analysis of network meta-analysis after excluding the four studies on single-agent chemotherapy. (A) Pooled ORs (95% credible intervals) for adverse events in the upper triangle and platelet transfusion in the lower triangle. (B) Pooled ORs (95% credible intervals) for thrombosis in the lower triangle. (C) Sucra for the sensitivity analysis on adverse events. (D) Sucra for the sensitivity analysis on platelet transfusion. (E) Sucra for the sensitivity analysis on thrombosis. (F) Bayesian ranking results of the sensitivity analysis on adverse events. (N) Bayesian ranking results of the sensitivity analysis on platelet transfusion. (O) Bayesian ranking results of the sensitivity analysis on thrombosis.

A.

**Placebo**					
0.75 (0.19,2.99)	**Avatrombopag**				
**3.50 (1.75,6.98)**	4.66 (0.99,21.91)	**rhIL-11**			
5.97 (0.83,42.92)	7.95 (0.71,88.61)	1.71 (0.27,10.83)	**rhTPO**		
**7.37 (1.50,36.18)**	**9.82 (1.19,80.96)**	2.11 (0.50,8.83)	1.23 (0.12,12.80)	**mIL-11**	
3.88 (0.46,32.66)	5.18 (0.41,65.64)	1.11 (0.12,10.42)	0.65 (0.04,11.87)	0.53 (0.04,7.53)	**Romiplostim**

B.

**Placebo**	1.14 (0.46,2.82)	1.20 (0.35,4.10)	**4.63 (2.74,7.81)**	**3.52 (1.13,10.93)**	2.90 (0.86,9.77)	1.54 (0.18,13.44)
0.88 (0.35,2.18)	**Eltrombopag**	1.06 (0.23,4.87)	**4.07 (1.43,11.61)**	3.10 (0.73,13.23)	2.55 (0.56,11.62)	1.35 (0.13,14.19)
0.83 (0.24,2.85)	0.95 (0.21,4.37)	**Avatrombopag**	**3.85 (1.01,14.67)**	2.93 (0.55,15.62)	2.41 (0.43,13.61)	1.28 (0.11,15.49)
**0.22 (0.13,0.37)**	**0.25 (0.09,0.70)**	**0.26 (0.07,0.99)**	**rhIL-11**	0.76 (0.28,2.08)	0.63 (0.21,1.88)	0.33 (0.04,3.09)
**0.28 (0.09,0.88)**	0.32 (0.08,1.38)	0.34 (0.06,1.81)	1.31 (0.48,3.59)	**rhTPO**	0.82 (0.19,3.64)	0.44 (0.04,5.04)
0.35 (0.10,1.16)	0.39 (0.09,1.79)	0.41 (0.07,2.34)	1.60 (0.53,4.78)	1.22 (0.27,5.38)	**mIL-11**	0.53 (0.04,6.38)
0.65 (0.07,5.67)	0.74 (0.07,7.74)	0.78 (0.06,9.42)	3.01 (0.32,27.94)	2.29 (0.20,26.39)	1.88 (0.16,22.59)	**Romiplostim**

C

**Placebo**	0.68 (0.20,2.30)	0.80 (0.18,3.53)	3.87 (0.41,36.33)
1.47 (0.40,5.38)	**Eltrombopag**	1.18 (0.17,8.02)	5.69 (0.45,72.75)
1.03 (0.09,11.66)	0.70 (0.04,11.02)	**Avatrombopag**	4.84 (0.33,70.95)
0.44 (0.04,4.48)	0.30 (0.02,4.29)	0.43 (0.01,12.35)	**Romiplostim**

D

**Placebo**	**0.50 (0.26,0.97)**	**0.32 (0.15,0.72)**
1.41 (0.68,2.92)	**Eltrombopag**	0.64 (0.23,1.81)
**2.08 (1.00,4.58)**	1.48 (0.50,4.33)	**Avatrombopag**

E

Placebo		
0.66 (0.09,4.77)	Avatrombopag	
**0.01 (0.00,0.22)**	**0.02 (0.00,0.60)**	Romiplostim

F

Placebo		
**4.30 (1.02,9.02)**	rhTPO	
**-0.40 (-5.66,-4.86)**	0.90 (-4.32,6.13)	rhIL-11

G

Placebo	0.78 (0.25,2.37)	0.43 (0.17,1.10)	**0.26 (0.07,0.91)**	0.89 (0.31,2.55)
1.29 (0.42,3.94)	Eltrombopag	0.55 (0.13,2.38)	0.33 (0.06,1.80)	1.15 (0.25,5.33)
2.33 (0.91,5.97)	1.81 (0.42,7.79)	rhIL-11	0.60 (0.26,1.40)	2.08 (0.51,8.51)
**3.87 (1.09,13.68)**	3.00 (0.56,16.22)	1.66 (0.72,3.86)	rhTPO	3.45 (0.67,17.84)
1.12 (0.39,3.22)	0.87 (0.19,4.04)	0.48 (0.12,1.98)	0.29 (0.06,1.50)	Romiplostim

H

Placebo				
**-23.40 (-42.97,-3.83)**	Avatrombopag			
**-28.44 (-46.67,-10.22)**	-5.04 (-31.79,21.70)	rhIL-11		
**-12.00 (-20.67,-3.33)**	11.40 (-10.01,32.81)	16.44 (-3.74,36.63)	rhTPO	
**-30.84 (-52.52,-9.17)**	-7.44 (-36.65,21.76)	-2.40 (-14.14,9.34)	-18.84 (-42.19,4.51)	mIL-11

I

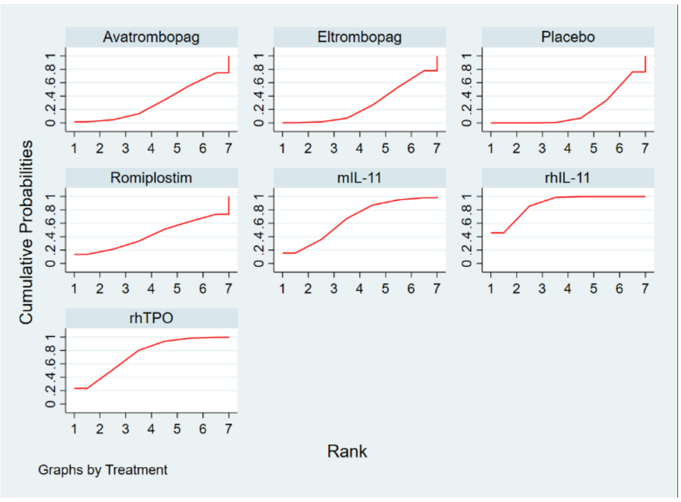

Adverse events

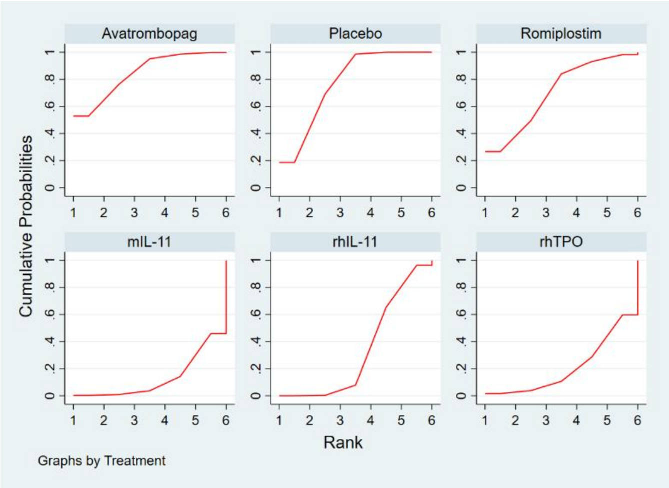

Platelet transfusion

J

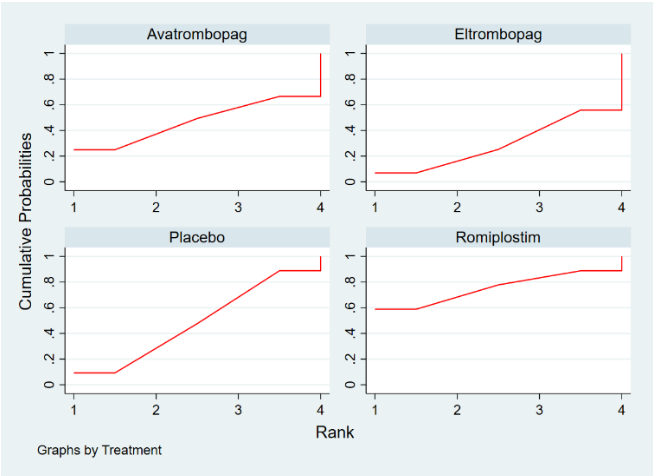

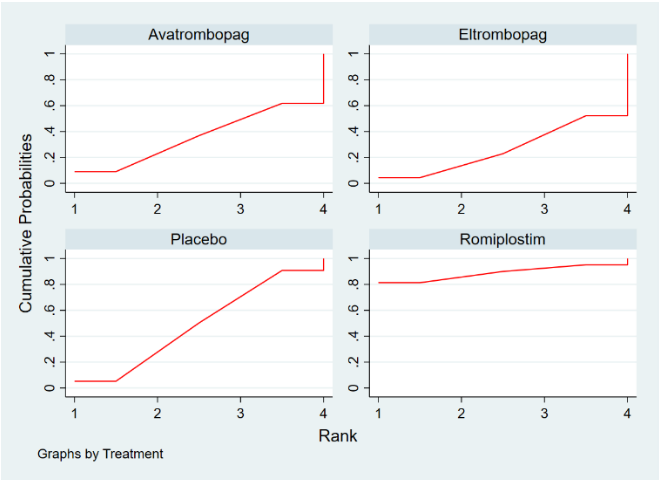

Thrombosis **Bleeding events**

K

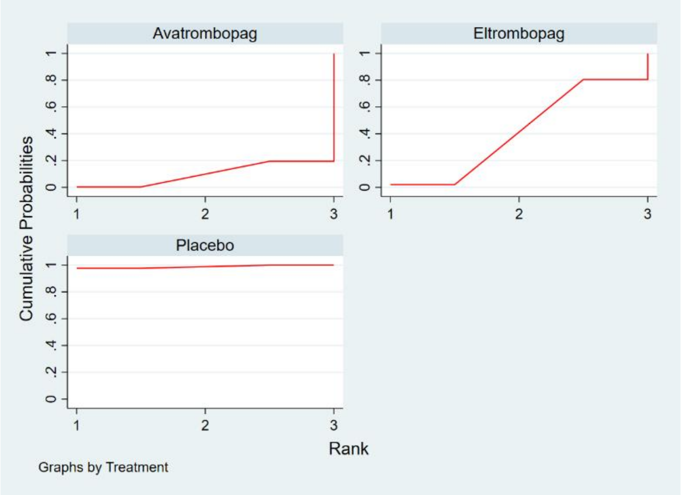

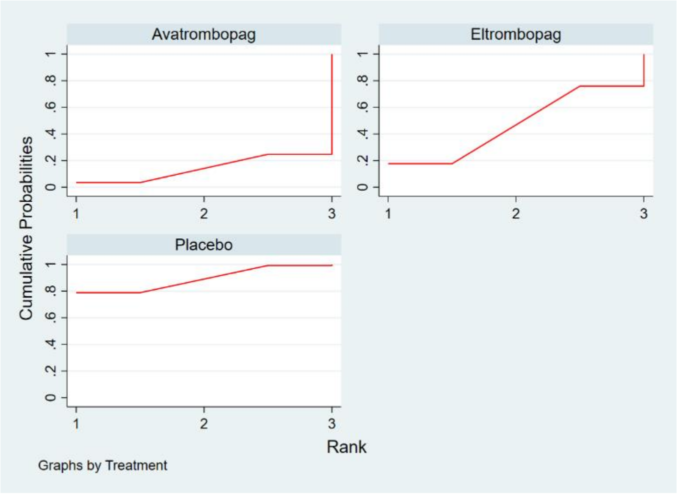

Neutropenia **Anemia**

L

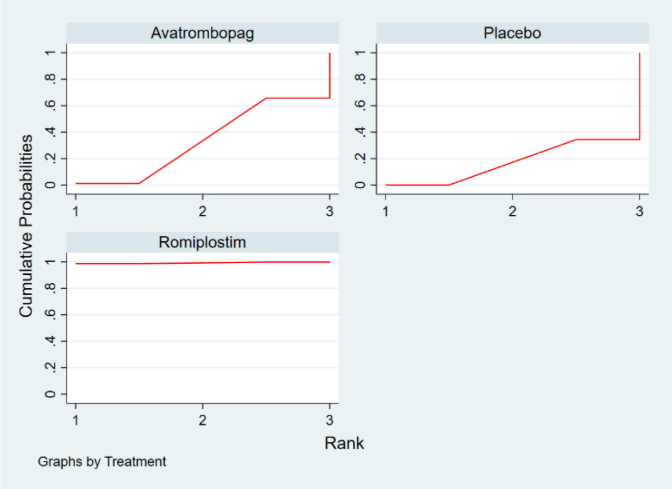

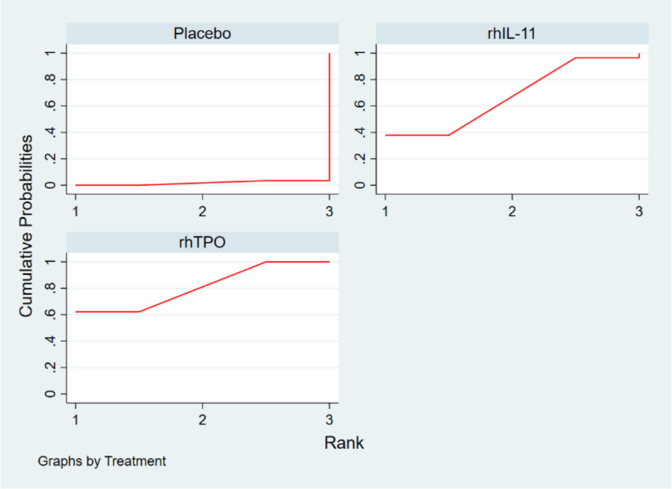

PLT recovery to ≥100×109/L **PLT recovery to ≥100×109/L(d)**

M

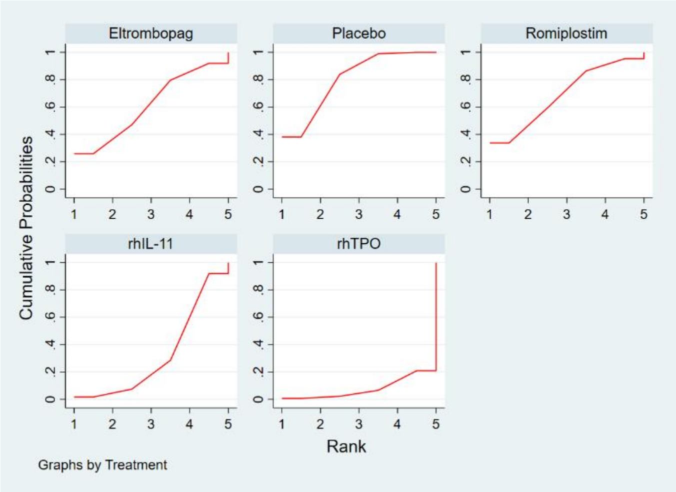

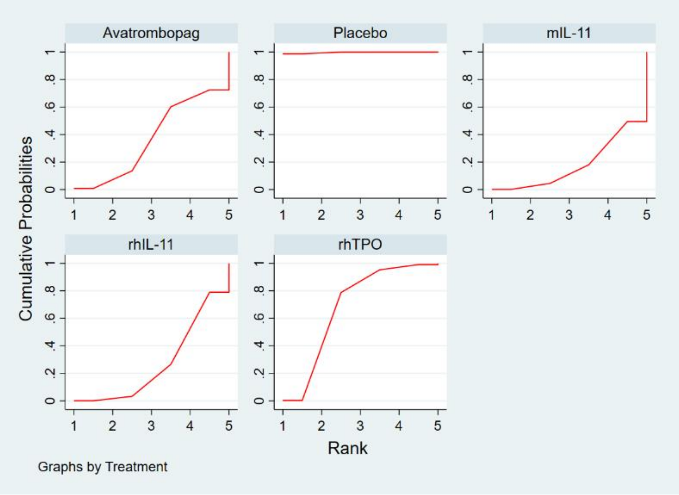

Grade 3/4 thrombocytopenia **Nadir platelet count**

N

Treatment	SUCRA	PrBest	MeanRank
Placebo	19.6	0.0	5.8
Eltrombopag	27.8	0.2	5.3
Avatrombopag	30.8	1.5	5.2
rhIL-11	88.3	45.7	1.7
rhTPO	74.4	23.4	2.5
mIL-11	66.5	15.6	3.0
Romiplostim	42.5	13.5	4.4

Adverse events

Treatment	SUCRA	PrBest	MeanRank
Placebo	77.2	18.6	2.1
Avatrombopag	84.6	52.9	1.8
rhIL-11	34.0	0.0	4.3
rhTPO	20.9	1.6	5.0
mIL-11	13.0	0.3	5.3
Romiplostim	70.3	26.7	2.5

Platelet transfusion

Treatment	SUCRA	PrBest	MeanRank
Placebo	48.6	9.3	2.5
Eltrombopag	29.3	6.9	3.1
Avatrombopag	47.0	25.0	2.6
Romiplostim	75.1	58.8	1.7

Thrombosis

Treatment	SUCRA	PrBest	MeanRank
Placebo	48.8	5.3	2.5
Eltrombopag	26.5	4.4	3.2
Avatrombopag	35.9	9.1	2.9
Romiplostim	88.8	81.3	1.3

Bleeding events

Treatment	SUCRA	PrBest	MeanRank
Placebo	98.8	97.7	1.0
Eltrombopag	41.3	2.1	2.2
Avatrombopag	9.9	0.3	2.8

Neutropenia

Treatment	SUCRA	PrBest	MeanRank
Placebo	89.1	78.8	1.2
Eltrombopag	46.8	17.7	2.1
Avatrombopag	14.1	3.5	2.7

Anemia

Treatment	SUCRA	PrBest	MeanRank
Placebo	17.2	0.1	2.7
Avatrombopag	33.5	1.2	2.3
Romiplostim	99.3	98.7	1.0

PLT recovery to ≥100×10^9/L

Treatm~t	SUCRA	PrBest	MeanRank
Placebo	1.7	0.0	3.0
rhIL-11	67.2	37.8	1.7
rhTPO	81.1	62.2	1.4

PLT recovery to ≥100×10^9/L(d)

Treatment	SUCRA	PrBest	MeanRank
Placebo	80.2	38.1	1.8
Eltrombopag	61.1	25.8	2.6
rhIL-11	32.4	1.7	3.7
rhTPO	7.6	0.7	4.7
Romiplostim	68.7	33.7	2.3

Grade 3/4 thrombocytopenia

Treatment	SUCRA	PrBest	MeanRank
Placebo	99.7	98.7	1.0
Avatrombopag	36.8	0.8	3.5
rhIL-11	27.2	0.0	3.9
rhTPO	68.3	0.3	2.3
mIL-11	18.0	0.2	4.3

Nadir platelet count

Supplementary Figure 9. Sensitive analysis of network meta-analysis after excluding the four studies on non-standard doses. (A) Pooled ORs (95% credible intervals) for platelet transfusion in the lower triangle. (B) Pooled ORs (95% credible intervals) for adverse events. (C) Pooled ORs (95% credible intervals) for thrombosis in the lower triangle and bleeding event in the upper triangle. (D) Pooled ORs (95% credible intervals) for neutropenia in the upper triangle and anemia in the lower triangle. (E) Pooled ORs (95% credible intervals) for PLT recovery to ≥100×10^9/L in the lower triangle. (F) SMD (95% credible intervals) for PLT recovery to ≥100×10^9/L(d) in the lower triangle. (G) Pooled ORs (95% credible intervals) for grade 3/4 thrombocytopenia. (H) SMD (95% credible intervals) for nadir platelet count. (I) Sucra for the sensitivity analysis on adverse events and platelet transfusion. (J) Sucra for the sensitivity analysis on thrombosis and bleeding events. (K) Sucra for the sensitivity analysis on neutropenia and anemia. (L) Sucra for the sensitivity analysis on PLT recovery to ≥100×10^9/L and PLT recovery to ≥100×10^9/L(d). (M) Sucra for the sensitivity analysis on grade 3/4 thrombocytopenia and nadir platelet count. (N) Bayesian ranking results of the sensitivity analysis on adverse events and platelet transfusion. (O) Bayesian ranking results of the sensitivity analysis on thrombosis and bleeding events. (P) Bayesian ranking results of the sensitivity analysis on neutropenia and anemia. (Q) Bayesian ranking results of the sensitivity analysis on PLT recovery to ≥100×109/L and PLT recovery to ≥100×109/L(d). (R) Bayesian ranking results of the sensitivity analysis on grade 3/4 thrombocytopenia and nadir platelet count.
